# View-tuned and view-invariant face encoding in IT cortex is explained by selected natural image fragments

**DOI:** 10.1038/s41598-021-86842-7

**Published:** 2021-04-09

**Authors:** Yunjun Nam, Takayuki Sato, Go Uchida, Ekaterina Malakhova, Shimon Ullman, Manabu Tanifuji

**Affiliations:** 1 Laboratory for Integrative Neural Systems, RIKEN Center for Brain Science, Wako-shi, Saitama, Japan; 2 Research Promotion Division, Fukushima University, Fukushima, Japan; 3 Lab. Physiology of Vision, Pavlov Institute of Physiology, Saint-Petersburg, Russia; 4 Department of Computer Science and Applied Mathematics, The Weizmann Institute of Science, Rehovot, Israel; 5 Department of Life Science and Medical Bio-Science, Faculty of Science and Engineering, Waseda University, Shinjuku, Tokyo Japan

**Keywords:** Cognitive neuroscience, Computational neuroscience, Visual system

## Abstract

Humans recognize individual faces regardless of variation in the facial view. The view-tuned face neurons in the inferior temporal (IT) cortex are regarded as the neural substrate for view-invariant face recognition. This study approximated visual features encoded by these neurons as combinations of local orientations and colors, originated from natural image fragments. The resultant features reproduced the preference of these neurons to particular facial views. We also found that faces of one identity were separable from the faces of other identities in a space where each axis represented one of these features. These results suggested that view-invariant face representation was established by combining view sensitive visual features. The face representation with these features suggested that, with respect to view-invariant face representation, the seemingly complex and deeply layered ventral visual pathway can be approximated via a shallow network, comprised of layers of low-level processing for local orientations and colors (V1/V2-level) and the layers which detect particular sets of low-level elements derived from natural image fragments (IT-level).

## Introduction

Primates have a fascinating capability to recognize faces regardless of variation in size, position, illumination, and view angle^1^. Among these variations, the capability to discern individual faces regardless of facial views is remarkable, considering the vast differences in pixel space created by view changes. The face neurons in the inferior temporal (IT) cortex are characterized by their selectivity to faces versus non-face objects, and act as the neural substrate enabling the view-invariant face recognition^2^. These neurons are also known for their view and identity tuning properties: not all but many of these neurons respond to particular views of faces, and their identity tuning at the preferred views is relatively broad^3,4^. Previous studies suggest that objects, including faces, are represented by combinations of activities of IT neurons^3,5–8^. In particular, Dubois et al. reported that faces from different identities are linearly separable in the space where each axis represents the response level of a face neuron to the face images with varying facial views^5^. Therefore, a good model of individual face neurons well explaining their view or identity tuning properties would give insights into our understanding of how the face neurons cooperate with each other to make identities separable.

However, we do not have well-established computational models of individual face neurons that explain their view and identity tuning properties so far. Yamins et al. discovered that the responses of IT neurons can be reproduced from a linear combination of activations collected from the higher layers of the deep convolutional neural network (DCNN)^9,10^. However, in DCNNs, intermediate processing between input and output is opaque. This “black-boxed” nature of DCNNs prevents intuitive interpretation of how the neurons are tuned to particular views and identities. Recently, Le Chang et al. reported that the visual features of face neurons were described by positions of the facial landmarks (the positions unique to faces, such as the eyes, nose, and mouth)^11^. However, we do not know whether this is the right model of the face neurons because how and where in the ventral visual pathway, these landmarks were extracted has not yet been explicated.

**Figure 1:**
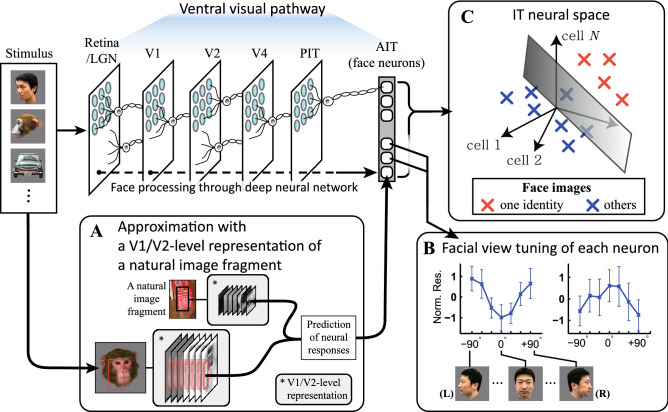
The view-tuned neurons in the inferior temporal cortex are regarded as the neural substrate for view- invariant face recognition, but we still do not fully understand the facial features detected by these neurons. Here, we approximated visual features encoded by these neurons as combinations of local orientations and colors (low-level representation), originated from natural image fragments. The visual features identified from the view-tuned face neurons showed that (1) IT columnar responses can be emulated by a shallow network detecting low-level representations derived from natural image fragments (**A**); (2) the resulting features can reproduce the preference of the neurons to particular facial views. Norm. Res: normalized responses. (**B**); (3) the faces of one identity can be separated from the faces of other identities in a space where each axis represents the view sensitive visual features, providing the evidence that the view-invariant face representation can be established by combinations of the view sensitive features (**C**). LGN: Lateral geniculate nucleus, PIT: posterior IT, AIT: anterior IT.

In the present study, we constructed a novel model using existing knowledge of visual information processing before it reaches the IT cortex. We hypothesized that the visual feature encoded by each face neuron might be characterized by the V1/V2-level representation of a specific natural image fragment by two reasons^12^. First, IT neurons are fundamentally dedicated to recognizing objects in natural scenes, therefore the visual feature of a face neuron can be approximated by a natural image fragment^7,13^. Second, IT neurons receive visual information preprocessed in V1/V2, therefore the feature of a face neuron can be given by the V1/V2-level representation of the fragment, rather than the representation in the pixel space^3,14^. In practice, we designed an artificial neural network with a shallow network structure (Fig. 1A), where the response of an individual face neuron to a stimulus was given by the Euclidean distance between a fragment assigned to the neuron and the stimulus in their V1/V2-level representations.

In a short summary, the resultant features well explained view and identity tuning of individual face neurons, and combinations of these features made view-invariant face representation possible. These results suggest that the visual information processing through the ventral visual pathway can be approximated by a shallow network (Fig. 1A) consisting of layers of low-level processing for local orientations and colors (V1/V2-level) and the layers which detect particular sets of low-level elements derived from natural image fragments (IT-level). The simple structure of the shallow network provided intuitive explanations for view-tuned and view-invariant face encoding of the face neurons in the IT cortex (Fig. 1B,C).

## Results

### Recording neural responses from columns tuned to particular facial views

**Figure 2:**
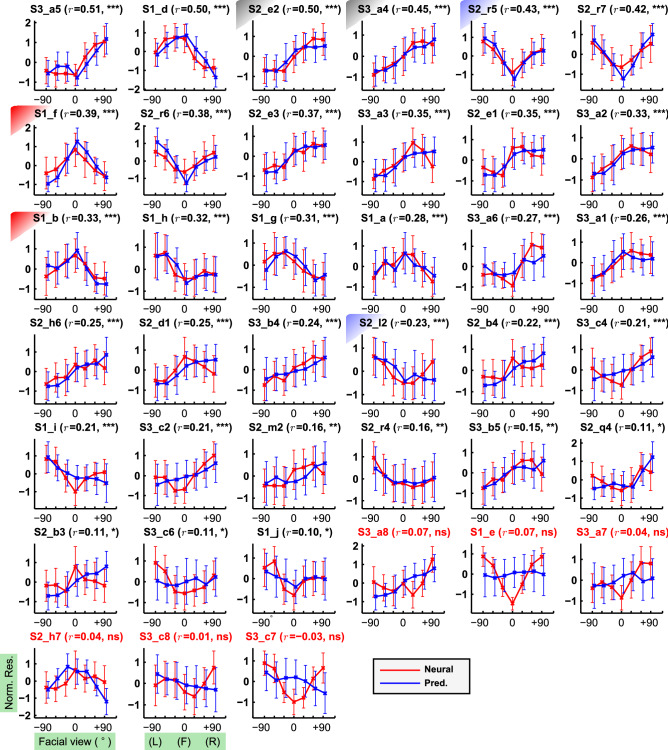
Actual and predicted view tuning curves from 39 recording sites. Mean and standard deviation across identities are plotted after taking a z-score (see “Methods”). The sites are sorted by the predictability ($r$), estimating the correlation between two view tuning curves. The site ID is labeled above each panel. Among 39 sites, significant correlation (p < 0.05) was found from 33 sites (= 84.6%, the site IDs are in black). *p < 0.05, **p < 0.01, and ***p < 0.001.

Neural responses were recorded for 1,509 object images (Fig. S1), consisting of view-controlled faces (n = 287), view-uncontrolled faces (n = 532), and non-face objects (n = 690). Since neurons that responded to similar visual features were clustered together in a functional column^4,15,16^, columnar responses evoked by these stimuli were recorded from 190 sites in the anterior IT cortex of three macaque monkeys (see SI text). Among the 190 sites, we selected reliable and face-selective sites (n = 88) based on tuning response repeatability (correlation > 0.5 between even versus odd trial-averaged object responses, with Spearman-Brown correction^17^), and face-selective index^18^ (> 1/3) of their responses (see SI Text). We further selected 39 sites that exhibited significant response variations across the facial views (p < 10^−6^, ANOVA test; see SI Text and Fig. S2). These sites revealed various view tuning patterns (Fig. 2, red lines), including preferences for frontal views (Fig. 2, S1_f, S1_b, denoted by the red shades), right profiles (S2_e2, S3_a4, gray shades), and mirror-symmetric views (S2_r5, S2_l2, blue shades).

### Using natural image fragments as visual feature candidates

We utilized a fragment-based approach to identify features encoded by the face columns^12^. We prepared a dictionary consisting of a massive number of natural image fragments for use as candidate features (see SI Text and Fig. S3). Each of the fragments in the RGB pixel space was converted to a set of seven images consisting of four local orientations (0°, 45°, 90°, and 135°) and three colors (red, green, and blue), and the set was termed “feature candidate.” The stimuli used for the neural recordings were also converted to a set of seven images (Fig. S4). Using the candidate as the feature of a face column, we located the sub-region of the stimulus that showed the minimum Euclidean distance with the candidate. This step accounted for the position invariance of the IT neurons^19,20^. Similarly, we took into account scale invariance by enabling the candidate to search for the optimal size of the sub-region within the particular range ${\varvec{b}}$ (see SI text)^19,21^. The value of the minimum distance was then transferred to the predicted response using a radial basis function. Finally, we selected the feature of each column from the candidates based on a correlation between the predicted and neural responses to the stimuli. The first correlation coefficient (global correlation, ${r}_{{\text{globa}}{\text{l}}}$) was calculated for the entire set of stimuli consisting of 532 faces and 690 non-face objects and the second coefficient (local correlation, ${r}_{\text{local}}$) was calculated only for the stimuli of 532 faces. Among the fragments with a significant result for both local and global correlation (α = 0.05, see SI Text), the candidate with the highest global correlation was selected as the feature of the column (“identified visual feature”). Two correlation coefficients were calculated, as the quantification of the entire set of stimuli (${r}_{\text{global}}$) was not sensitive enough to capture variation among the subset of the stimuli.

**Figure 3:**
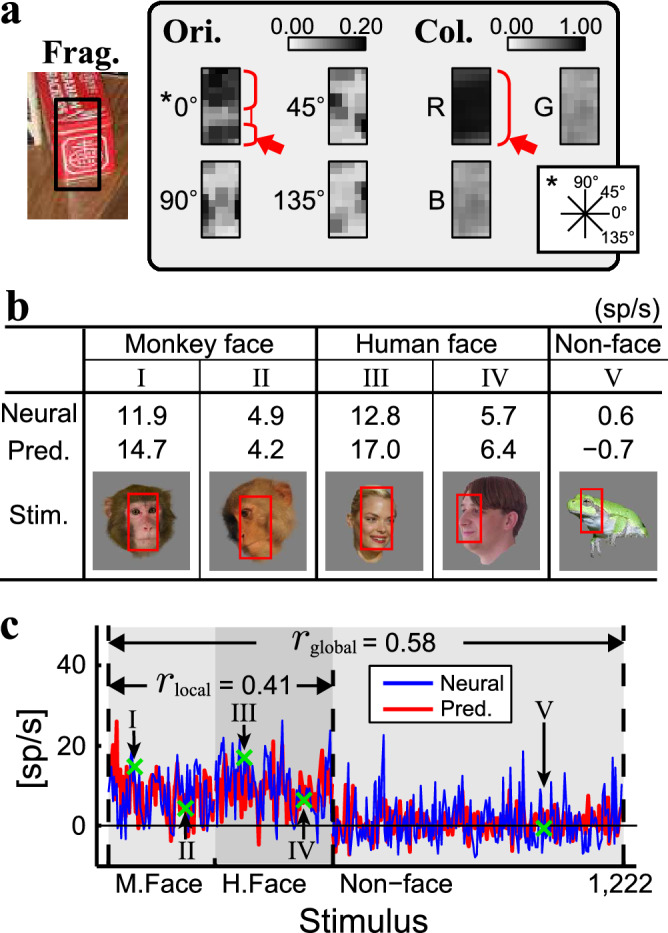
Example feature candidate found to describe the neural responses recorded from site S1_a. (**A**) This feature candidate was originated from the fragment of a red snack box image (black rectangle). “Ori.” and “Col.” refer to the orientation and color channels, respectively. (**B**) Five example stimuli whose neural responses were located in the first and third quartiles among the monkey faces (“I” and “II”), the same quartiles among the human faces (“III” and “IV”), and the median among non-faces (“V”). The predicted responses for these stimuli are denoted by green “x” marks in (C). In the images, the red rectangles denote the sub-region that showed the best match with the feature candidate. The numbers indicate neural (above) and predicted (below) responses. sp/s: spikes/s. (**C**) The neural response from site S1_a (red line), and predicted responses generated from the candidate (blue) (vertical axis) plotted against stimuli grouped by stimulus categories (horizontal axis). M.face, H.face, and Non-face indicate categories of monkey faces, human faces, and non-face objects, respectively. Please note that the predicted responses ranging from 0 to 1 were converted to spike rates using the mean value of the face responses and of the non-face object responses (see “Methods”).

Figure 3A depicts the identified visual features for site S1_a in this way. The feature was characterized by a combination of reddish color and horizontal orientation components (Fig. 3A, arrows), which coincided with the facial configuration of the eyes, nose, and mouth (Figs. 3B and S4). This feature matched the faces (stimuli I, II, III, and IV in Fig. 3B) more readily than the non-face objects (stimulus V; see also Fig. S4). Thus, the feature predicted higher responses to faces (8.91 ± 4.95 sp/s; n = 532) than to the non-face objects (0.64 ± 5.60 sp/s; n = 690). These predicted responses were significantly correlated with the neural responses (${r}_{\text{global}}$ = 0.58, n = 1222, *p* < 10^−6^) (Fig. 3C).

Face selectivity (neural response preference for face over non-face objects) was the defining property of the face columns, but individual face columns were uniquely characterized by tuning the variations across the faces. For example, site S1_a responded better to some faces (stimuli I and III in Fig. 3B) than to the other faces (stimuli II and IV). The identified feature also explained the site-specific variation in the faces. For example, stimuli I and III had facial configurations with strong horizontal components (Fig. S4) that readily matched the candidate, while the other stimuli had tilted (stimulus II) or narrow (stimulus IV) facial areas that reduced the matching capacity. Thus, significant correlation between the predicted and neural responses across faces was found (${r}_{\text{local}}$ = 0.41, n = 532, p < 10^−6^).

### Validation of identified visual features

**Figure 4:**
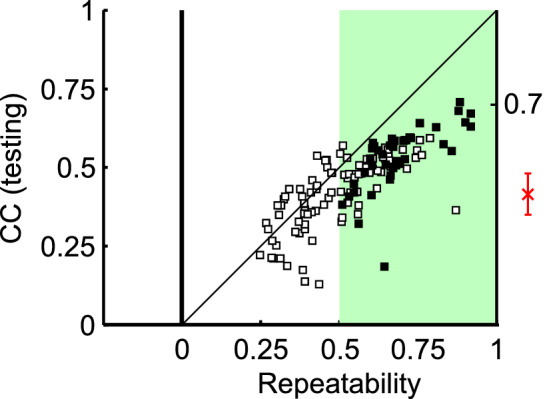
The vertical axis, view-uncontrolled face, and non-face object stimuli (n = 1222) are split into two halves for the two-fold cross-validation test. For each site, the visual feature identified from responses to one half is utilized to predict responses to the other half, and the correlation coefficient between the neural and predicted responses was used as the performance measure. The horizontal axis, the repeatability of each face column estimated by the correlation between even and odd trial-averaged object responses (Spearman-Brown correction). Among 152 face-selective columns (FSI > 1/3, rectangles), we selected 88 reliable sites (repeatability > 0.5) to conduct the cross-validation test. We further identified 39 view-tuned sites (black rectangles) and predicted their view tuning curves (Fig. 2). The red error bar is the performance for predicting activations of a higher DCNN layer (see “Discussion”).

This study used two methods to determine how well an identified visual feature could reproduce a site’s neural responses. First, a standard cross-validation test was applied. We  divided the view-uncontrolled faces and non-face objects (n = 1,222) into equal-sized training and test sets (n = 611). Visual features were identified using neural responses to the training set for the sites that revealed reliable and face-selective neural responses (n = 88; the shaded area in Fig. 4). Then, the visual features were utilized to predict responses to the test set, and the prediction performance, cc, was assessed. For the test set, the average correlation coefficient between the predicted and neural responses was 0.51 ± 0.09 for ${r}_{\text{global}}$ and 0.37 ± 0.12 for ${r}_{\text{local}}$ (p < 10^−6^ for both of ${r}_{\text{global}}$ and ${r}_{\text{local}}$). We compared this performance to the repeatability of the neural responses (= 0.65 ± 0.11; Spearman-Brown correction) estimated by the correlation coefficient between the trial-averaged tuning responses on the odd versus even trials (Fig. 4). The ratio between the cross-validation performance (${r}_{\text{global}}$) and the repeatability was 81.0%. The measure of explained variance ratio was 65.7%. The comparison of this ratio with previous studies is not straightforward since conditions are different from study to study. The ratio in our study was higher than the ratio in a previous method using DCNN (48.5%)^9^. However, neural responses of their study was based on both face and non-face neurons, while we restricted our analysis to face columns. Another study gave the ratio of 80.0%^11^. However, their stimuli included only faces in contrast to our study using both faces and non-face objects.

**Figure 5:**
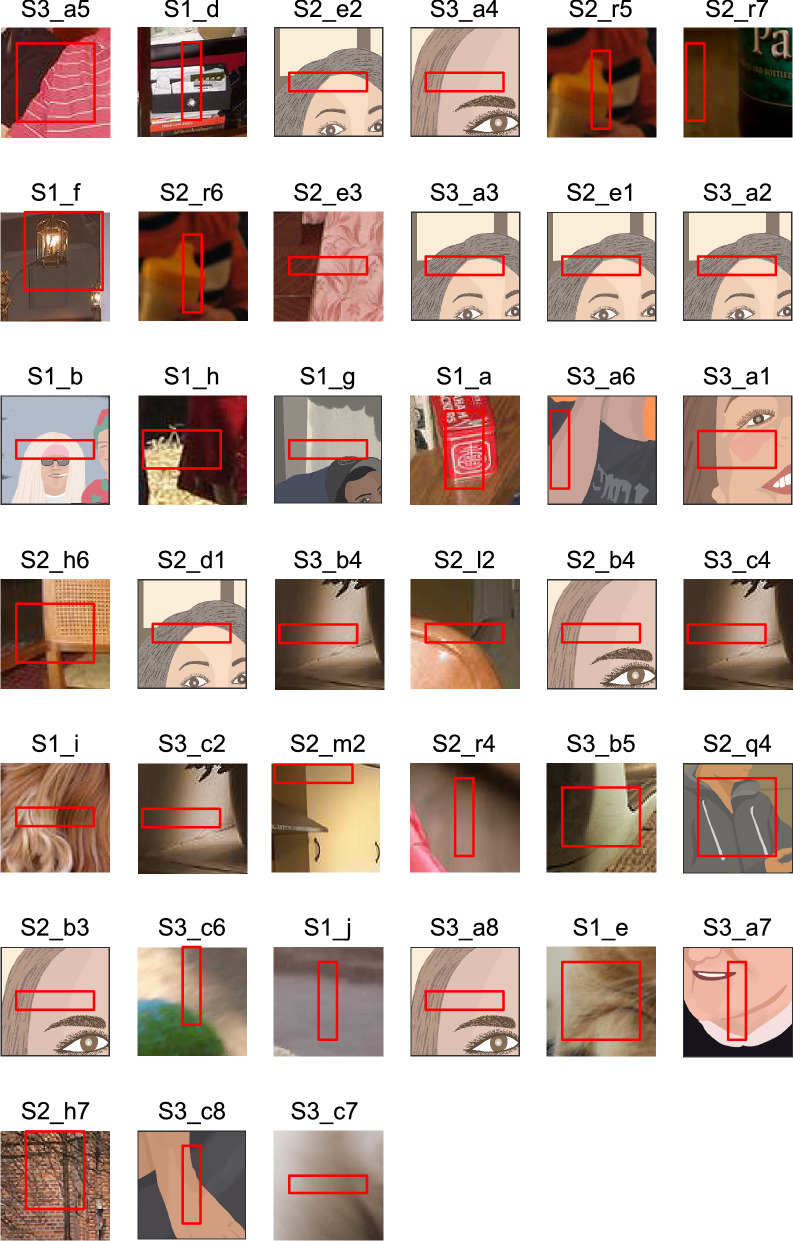
The fragments where features of 39 recording sites were originated. In each panel, the rectangular region demarcated in red indicates the fragment. The fragments are arranged in the same order as the tuning curves shown in Fig. 2. Although the features are represented in the four-orientation and three-color space, we can intuitively understand how these fragments provide a particular view tuning property. For example, sites S3_a5 (the leftmost fragment in the top row) and S2_e3 (the third fragment from the left in the second row) reveal the left side to be dark and right side to be bright that are consistent with the preference of these sites to the right profile faces. The features of the sites tuned to frontal faces or tuned to the left and right profile faces tended to have complex features (sites S1_a, S1_f). Please keep in mind that all of these features explained the general preference of these sites to faces over non-face objects (Figs. 3C, 4). We observed the same feature identified for multiple sites. For example, the feature of site S2_e2 was also identified in the sites (S2_e1 and S2_d1) recorded from the same monkey and also in the sites recorded from another monkey (S3_a3 and S3_a2). The result suggests that there is a canonical feature set in high-level vision (see SI for the detail). Please note that the fragments including faces are replaced with illustrations because of the copy right regulation of Scientific report. See the SI text for the online link to the figure with the original fragments. The SI text also gives the online link to the top five fragments for each site.

**Figure 6:**
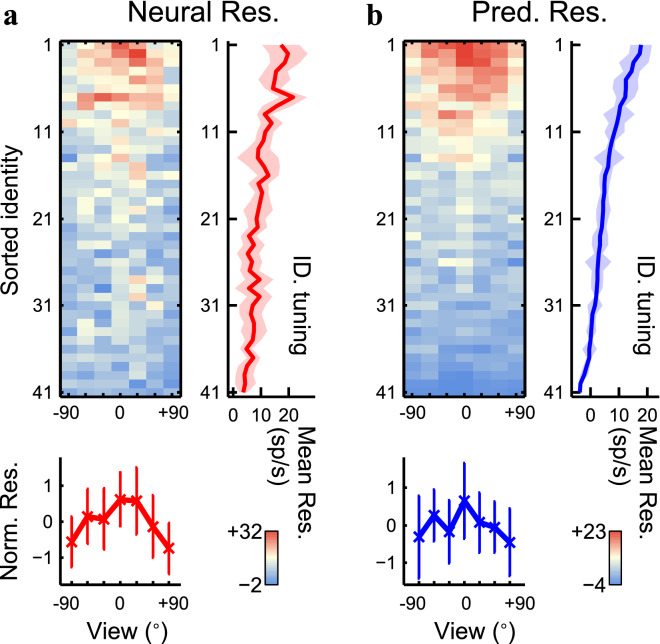
(**A**) The neural responses of 287 view-controlled face stimuli recorded from site S1_a. The vertical axis, 41 identities in descending order of the predicted responses. The horizontal axis, 7 views from left (− 90) to right (+ 90) profiles taken by every 30°. (**B**) The predicted responses generated from the feature candidate shown in Fig. 3A. In both panels, the trace in the right side indicates the identity tuning curve obtained by averaging across views, and the trace at the bottom indicates the view tuning curve obtained by averaging responses normalized with the z-score (see “Methods”). The shades and error bars indicate the standard deviation.

Secondly and more critically, for the recording sites that revealed significant view tuning (n = 39), we examined the ability of the identified visual features to predict the response properties unique to specific face columns. The visual features for each of these columns were identified by using their neural responses to view-uncontrolled faces and non-face objects (Fig. 5; SI text also gives the link to the features identified from 49 sites with no significant view tuning). Then, the identified features were used to predict the responses for the view-controlled faces (n = 287, taken from seven facial views of 36 human and five monkey identities). For example, site S1_a revealed a preference for the frontal view (Fig. 6A, bottom). Responses predicted from the identified features (Fig. 3A) also showed a preference for the frontal faces, and the correlation between the two view tuning curves (the bottoms of Figs. 6A,B) was 0.281 (p = 1.3 × 10^−6^, n = 287) (see the predictability in SI text for this correlation measure). Site S1_a also revealed broad identity tuning (Fig. 6A, right). The identified features accurately predicted identity tuning (Fig. 6B, right), and the Spearman’s correlation coefficient between the predicted and neural responses to identities was 0.902 (p < 10^−6^, n = 41).

We repeated the same analysis for other sites to examine the ability of the identified features to predict the view and identity tuning properties. Among the 39 sites, 33 sites (= 84.6%) showed a significant correlation (p < 0.05) between the neural and predicted view tuning curves (Fig. 2). This result indicated that the identified visual features predicted the view tuning curves despite the absence of view-controlled faces in the stimulus set used to identify this feature. Fourteen out of 39 sites showed significant response variation across the face identities (*p* < 10^−6^, ANOVA), and significant Spearman’s correlations (*p* < 0.05) were observed from 13 sites (92.9%; see Fig. S6). This indicated that the identified visual features also accurately predicted identity tuning to novel identities that were not included in the stimulus set used to identify the features.

### Ability of the identified visual features to quantitatively explain facial view tuning

**Figure 7:**
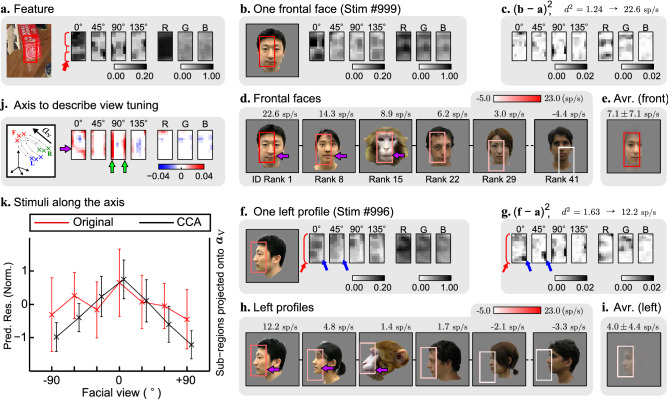
(**A**) The identified visual feature of site S1_a. (**B**) The sub-region (red rectangle) of a front-face with the highest similarity to the feature. (**C**) The response was predicted from the squared distance between the feature (**A**) and the sub-regions (**B**) in local orientation and color components. (**D**) The sub-regions (rectangles) from the other front-faces. Above, the predicted responses for each face. Below, the ranks of faces with respect to the predicted responses to front-faces. (**E**) The weighted average of sub-regions captured from all 41 front-faces (see Supplementary Fig. 5 for the procedure). (**F**)–(**I**) The same as (**B**)–(**E**), but with the left profile faces of the same identities. The rotation from front to left view evokes two major changes: (1) the decrease of horizontal components (0°) (red arrows in (**F**) and (**G**)), and (2) decrease of orientation components at the lower right corner (blue arrows). These changes caused the squared distance (${d}^{2}$) to change from 1.24 to 1.63. The sub-region captured from different faces were almost the same regardless of views, although the weighted average image was faint in the left profile because of weaker predicted responses, as shown in (**E**) and (**I**). (**J**) The axis (${{\varvec{a}}}_{{\varvec{V}}}$) extracted from CCA to visualize how each component changes when the face was rotated from the non-preferred (left and right) to the preferred (front) view. (**K**) Inner products between the sub-regions of faces (n = 41 × 7) and the axis extracted from CCA (black line, right axis) plotted on top of the original view tuning curve (red line, left axis). Error bar, standard deviation across identities. The correlation coefficient between the two lines was 0.212 (*p* < 0.001), suggesting that the tuning curve was correctly approximated by using the axis.

An advantage of the fragment-based approach is that the identified features provide a quantitative explanation for the tuning property. For example, the identified feature of site S1_a (tuned to front-faces) consists of multiple local components (Fig. 7A) that matched the eyes and mouth in the front-face (Figs. 7B,C). These components were also prominent in the left profile (Fig. 7F), and the site captured part of a profile face where the eye and mouth were centered similarly to those in the front-face. However, the strengths of these local components were different from those in the front-face (arrows in Figs. 7F,G), resulting in a lower response to the left profile (= 12.2 sp/s) than to the front-face (= 22.6 sp/s). The complexity of this feature enabled the site to detect the same part of faces for different identities (Figs. 7D,E,H,L; see SI Text and Fig. S7 for the process to visualize the detected parts of faces) and the changes of the predicted responses between the front-face and the left profile were consistent except for the faces with weak responses regardless of the views such as rank 41 identity. The predicted responses across 41 identities were 7.1 ± 7.1 sp/s in front-faces (Fig. 7E) and 4.0 ± 4.4 sp/s in the left profile (Fig. 7I). A set of components explaining the response changes across all seven views for 41 identities was approximated with a single axis in the local orientation and color space extracted by the canonical correlation analysis (CCA) (see SI Text and Fig. S8). The resulting axis was then characterized by changes in the horizontal local orientations (Fig. 7J, purple arrow) involved with lip shapes in two different views (see purple arrows in Fig. 7D,H). We also found that horizontal rotation of the faces produced changes in the local orientations that are more substantial in the vertical edges than the center (Fig. 7J, 0°, 45°, 90° channels, see green arrows).

The mirror-symmetric view tuning curve observed from site S2_r5 was explained by a relatively complex feature consisting of multiple local components (Fig. S9). This feature was characterized by local orientations demarcated by broken red lines. These characteristics were found in both the left and right profile faces where the orientation components matched the region around the hairline and the region around the neck. However, the site did not respond well to front-faces because of the local orientation components derived from the eyes, nose, and mouth that occupied the central part of the sub-region detected by the feature (Figs. S9J,K, arrows). This result is also supported by quantitative analysis with CCA. These examples provided the very first evidence of representation using configural features at the neuron level.

In addition to these features consisting of multiple local components, there were sites representing a relatively small number (= 0 or 1) of local components. For example, the feature of site S2_e2 was characterized by the rectangular region that was separated into left and right parts by 45° to 90° local orientations, and these two parts were colored black in the left and skin color in the right parts (Fig. S10). Because of the characteristics of this feature, the most preferred stimulus was the right profile of a face where the captured region was partly covered by hair (Figs. S10B,C). The site did not respond to the left profile faces because the contrast of hair and skin was opposite (Figs. S10F,G). The preference for the right profile faces was preserved across the identities despite of the difference in captured regions. For example, the hairline around the cheek was captured in the most preferred face, but the hairline around the forehead was captured in the rank 15 face (Figs. S10D). The axis from CCA confirms that the hairline with the specific color arrangement was the reason for the higher responses in the right profiles.

In summary, the features identified by our fragment-based approach were able to quantitatively explain facial view tuning properties. Although detailed explanations for view tuning were different from site to site (see SI Text and Fig. S11 for additional examples), both simple and complex features contributed to favorable response predictions. The complex features, which could be termed “configural features,” consisted of multiple local components that allowed the site responses to capture the same face parts across views and identities (sites S1_a and S2_r5). On the other hand, there were features consisted of a relatively small number of local components. Each of these features could not specify a particular part of faces; thus, the position of the captured region of the faces was varied across different views and identities (sites S1_b and S2_e2). This study termed these features “local features.”

### Does the combination of the view-tuned face columns make view-invariant face representation possible?

**Figure 8:**
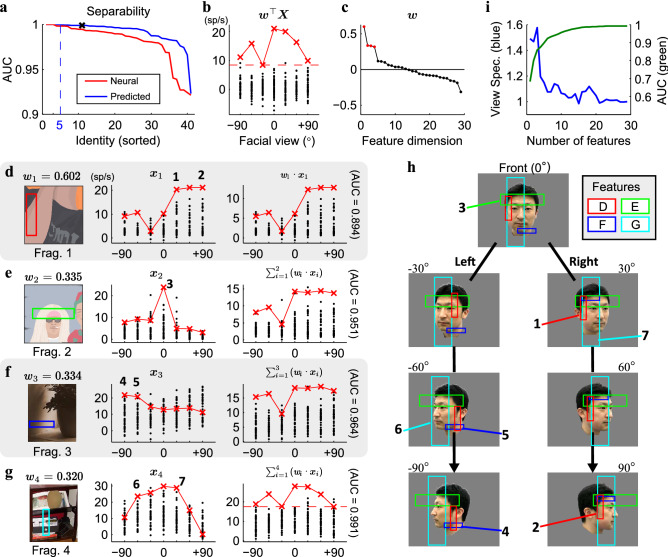
(**A**) The area under the ROC curve (AUC, vertical axis) for all pairs of one target identity and other 40 non-target identities (horizontal axis), evaluating how well target identities are separable from non-target identities in the space spanned by the predicted (blue) or neural (red) responses. (**B**) The seven faces of one target (red; see (H) for face images) and 280 faces of non-targets (black dots) represented along the axis ${\varvec{w}}$ (AUC = 0.998, see the black “x” in (**A**)). Here ${\varvec{w}}$ is the axis that maximizes the separability in the space of the predicted responses. (**C**) The values of each element in ${\varvec{w}}$ sorted in descending order. To search for key visual features that contribute to separation, we selected four elements with the largest absolute values (red dots). The features corresponding to these four elements are shown from (**D**) to (**G**). (**D**) The image fragment for the first visual feature (left) and its predicted responses to the faces of the target (red) and non-targets (black) identities (center). The responses are selective for right profiles (points 1 and 2) of the identity because of its good match with his right sideburn (areas 1 and 2 in (**H**)). (**E**) The second feature specific to the front-face (point 3) for being matched with the hairline (area 3). (**F**) The third feature is selective to the left profiles (points 4 and 5) for being matched with the left chin (areas 4 and 5). (**G**) The fourth feature is broadly tuned to the front views (points 6 and 7) for being matched with horizontal components in the front-faces (areas 6 and 7). As the number of associated features increases (right side of (**D**) to (**G**)), their linear combination becomes more specific to the target identity (AUC values increases from 0.894 to 0.991). (H) The sub-regions of the target faces matched with each feature. (**I**) View specificity (the ratio of the variance of seven target faces to the variance of all faces, normalized to set the last values to 1.0, blue) and AUC values (green) for 41 identities are averaged by increasing the number of features used for the linear combination (horizontal axis). Please note that a fragment including a face is replaced with an illustration because of the copy right regulation of Scientific report. Please refer to the SI text for the online link to the figure with the original fragments.

The critical question for view-invariant face recognition is whether combinations of activity for the view-tuned columns can make the face representation view-invariant. To address this question, we divided 287 faces (7 views × 41 identities) into seven faces from one target identity and 280 faces from 40 non-target identities. Next, the faces of the target identities were evaluated to determine if they were separable from the non-target faces in the feature space where each axis was defined by the predicted responses of the visual features. Here, the dimensions of the feature space were 29, despite identifying features from 39 view-tuned sites. This was because the identified features from multiple sites were the same, and duplicated features were not considered to be part of a different axis (Fig. 5). We searched for the projection vector ${\varvec{w}}$ that maximally separated the two groups of faces in the feature space, using simple linear regression. The separability achieved by ${\varvec{w}}$ was evaluated by AUC (the area under the receiver operating characteristic curve). For five out of 41 identities, the faces of a target identity were perfectly separated from the non-target faces (AUC = 1.0), and the average AUC value for 41 identities was 0.992 ± 0.014 (minimum value = 0.923; see Figs. 8A and S14). The generalization performance was tested using the leave-one-view-out test. Forty-one faces from a particular view were set aside as the testing set. Then, the ${\varvec{w}}$ was searched for using the training set that consisted of six target and 240 non-target (= 6 views × 40 identities) faces from remaining six views. Then, whether ${\varvec{w}}$ could correctly pick out the target face from the testing set was determined (see SI Text). The ratio for successful identification was measured as 65.9% (chance level = 1/41 ≈ 2.4%).

From the identities that were well separated from the others (for example, the identity depicted in Figs. 8B–H, AUC = 0.998), the key visual features that accounted for the separation were sought (Fig. 8B). This study focused on four of the 29 features that revealed the largest absolute values of the elements in ${\varvec{w}}$ (Fig. 8C). These features were selective to different facial views of the target identity (the middle column in Fig. 8D-G). When these features were linearly combined one by one, the AUC value progressively increased, and the view invariance improved (the right column in Fig. 8D-G). This was also the case for the other target identities (Fig. 8I). The number of features required for the mean AUC values to reach 0.9 was 6, which supported the idea that object representation was sparse. The features involved in the view-invariant face representation were different from identity to identity (Fig. S13A). In this way, view-invariant face representation was successfully established.

One notable advantage of the fragment-based approach was that the facial regions captured by all features were visible both in their positions and shapes (Fig. 8H). This advantage enabled the specification of facial regions captured by face columns in order to characterize identity. For example, the right sideburn (area 1 and 2 in Fig. 8H), hairline in the front view (area 3), left chin (area 4 and 5), and face region occupied by horizontal local orientations (area 6 and 7) were found to be critical in order to discriminate this identity. The common characteristics of facial regions captured by the 29 visual features (Fig. S13) will be further deliberated in the following discussion section. The existence of hyperplanes separating one identity from all others showed that view-invariant identity selectivity could be established by a linear combination of inputs tuned to multiple views^22,23^.

## Discussion

This study revealed that combinations of local orientations and colors originated from natural image fragments gave a good model of features encoded by view-tuned face columns. The resultant features reproduced responses to object images. More specifically, these features were tuned to particular facial views, and these tuning properties coincided well to those of face neurons. We also found that faces of one identity were separable from faces of other identities in a space where each axis represented one of these features. These results provided direct evidence that the view-invariant face representation in the brain can be achieved by combining view sensitive visual features.

### Facial features detected by the face columns

The fragment-based approach allowed us to find the facial features detected by the face columns and provided insight into the types of elements used by the brain to reconstruct faces (Figs. 8, S11, and S13). First, against a naïve thought, we could not find any evidence that face columns explicitly detected each of the facial parts such as the eyes, nose, and mouth. Instead, we could categorize the identified features into two types: local features (n = 17) and configural features (n = 12; see the features denoted by “*” mark in Fig. S13B). The local features consisted of a relatively small number of local components, and therefore each of these features could not capture particular facial features. The features often detected part of the hairline and face line, but exact positions were varied across views and identities (Fig. S10). The detections of the hairline and face line by these features may provide the neural basis for psychological studies showing that hairline is one of the critical factors required to identify faces^24^. On the other hand, the configural features consisted of relatively large numbers of local components that made the features to be matched with the same set of facial features regardless of views and identities. The captured parts of faces typically included eyes, nose, mouth, and hairline. Previous psychological studies suggested that the relational information of facial features is important in face recognition^25^. The finding of the columns encoding the configural features may provide the neural basis for these studies. Second, both of orientation and color components were necessary to predict responses of view-tuned face columns. One may feel this statement odd since color information does not largely contribute to explain specific view tuning (Fig. 7). However, the features of these columns were required to explain response variance across faces of different identities. In fact, if we extract the best feature from feature candidates lacking color components (*α* = 1.0), the extracted features only poorly explained the variance of neural responses across faces (Fig. S5). Involvement of color components could be crucial for representing faces of one identity separable from others in the space where axes are defined by activity of these columns since each identity can be characterized by specific colors and brightness which is more or less invariant across views but local orientation components are not. Finally, regardless of the types, the majority of the visual features of the face columns (69%) was originated from the non-face natural images. Although one may consider that features for face recognition should be found as a part of the face images, our results revealed that the features of the face columns were essentially generic (Fig. 3). The existence of the features derived from the non-face objects might explain face pareidolia^26^.

### Approximation of the deeply stratified ventral visual pathway with a shallow network

Each of the identified features was represented as a point in a local orientation and color space (V1/V2-feature space), where the axis represented the activity levels of the neurons in the early visual cortices, V1 and V2. In other words, the visual feature of each face column in the IT cortex was approximated by a set of outputs from neurons in the early visual cortices. The stimuli were also plotted in the same space, and the responses of the face column to the stimuli were calculated with Euclidean distances between the feature and the stimuli. Therefore, the shape of the manifold from a set of stimuli within this space determined the tuning properties of the column to these stimuli. The feature and manifold of the stimuli enabled mathematical explanations for the variance in responses of face columns.

Face images of one identity could be linearly separated from the other 40 identities in the high dimensional space where each axis represented the activity levels for each of the face columns (IT feature space; Fig. 8). The separability achieved with the predicted responses (the mean of AUC = 0.992 ± 0.014) was higher than the separability found with the real neural responses (Fig. S12; the mean of AUC = 0.981 ± 0.023). The existence of hyperplanes that separated one identity from all others revealed that the linear combination of the view-tuned features established a view-invariant identity representation.

Thus, this study suggested that the deeply stratified ventral visual pathway was well approximated via a shallow network that consisted of layers of convolution and local max pooling at V1/V2 and convolution and global minimum pooling at the IT cortex. Convolution kernels at the IT cortex corresponded to the features identified from the natural image fragments, and the global minimum pooling layers corresponded to the template matching with each of the stimulus. The prediction performance for each of the face columns was evaluated via the correlation coefficient between the predicted and neural responses; it increased with repeatability and tended to saturate at the level of 0.7 (Fig. 4). Perhaps, the reason for this performance saturation was that we modeled the ventral visual pathway with a shallow feedforward network architecture, and it is possible that other architectures such as recurrent neural networks can provide stronger predictions^27^, including better consistency with trial-by-trial response covariations^28^ Even with this limitation, we still could explain up to 50% of the variance in the object responses with our model. Thus, our novel method provided a valuable way to approach the mechanisms of how the ventral visual pathway works for view-invariant face processing.

### Contribution of our fragment-based approach to the field of deep convolutional neural networks (DCNN)

Recently, multiple studies have compared the DCNNs to the primate ventral visual pathway^9,29^. Yamins et al.^10^, for example, revealed that linear combinations of outputs of a DCNN coincided favorably with the responses of the IT neurons. Therefore, the approach taken in our study could also be applied the “artificial” deep neural networks, such as DCNNs, and would make approximation of DCNNs with shallow networks possible. In our preliminary experiment, which attempted to identify visual features that emulated the node activations of a higher DCNN layer (VGG-16, the thirteenth layer^30^; see SI text), the predicted responses from the identified visual features showed 0.420 ± 0.130 of correlation (max: 0.717, p < 0.05 in 510 among 512 sites; Fig. 4, the red cross with an error bar) with the VGG activation levels.

This preliminary result suggested that the fragment-based approach can provide a promising way to make a deep network smaller and faster by approximating the deep network structure into a shallow network^13,21^. Recently, the idea of compressing the deep layered networks into shallow networks drew attention to this area of research^31–33^. This issue was especially critical in the field of engineering, where attempts to embed deep neural networks into mobile devices or home appliances have not yet been fully realized^34^. Secondly, and more importantly, our method might elucidate the features encoded into the higher layers, as it did for face columns. Identification of these features could provide a breakthrough in realizing “explainable AI.” Despite great success using DCNNs for various object recognition tasks, the “black boxed” nature of these networks causes people to be reluctant to apply DCNN to safe-critical tasks^35^. Concern regarding the opaqueness of surfaces has been a serious issue for artificial intelligence or the machine learning field, and in-depth discussions about this issue are taking place in the name of “explainable AI” or “interpretable machine learning” (for example, the NIPS 2017 symposium on interpretable machine learning). In our next study, we will apply our method to each layer of the artificial deep neural network to validate how this opaqueness issue could be dealt with in the identified features.

## Methods

### Columnar response recording

Columnar responses were recorded from the three anesthetized Japanese macaque monkeys (*Macaca fuscata*). The surgical procedures and recording experiments were conducted as in the previous studies^16,36^. The experimental protocol was approved by the Experimental Animal Committee of the RIKEN institute and followed the guidelines of the RIKEN institute and the National Institutes of Health. During recordings, stimulus images were presented for 100 ms to the animals, and the size of the stimulus images was 200 × 200 pixels (20° × 20° in visual angle). The columnar responses of the site were calculated by averaging eight multi-unit activities that were recorded along the axis perpendicular to the cortical surface. The number of recording sites from the three monkeys were 33 (S1), 134 (S2), and 24 (S3), respectively.

### Fragment-based analysis

To search for the visual features that approximated neural responses of the face and non-face objects, we generated a massive number of feature candidates from natural image fragments^12^. This method began using 7753 natural images collected from the VOC 2010 image database^37^. To emulate the visual information processing conducted in the lower visual areas, the pixel images were preprocessed with the Gabor filter, and local max operation to obtain a V1/V2-level representation. Then, the preprocessed images were cut into 560,000 fragments, whose pixel size varied from [8 × 8] to [20 × 20]. These fragments were termed the “feature candidate,” and assumed that the visual feature of a target site could be found among these candidates.

Then, each candidate was utilized to generate a response vector where each element represented predicted responses to one of the stimuli. For each pair of a candidate and stimulus, the predicted response was calculated by allowing the stimulus image to pass through an artificial neural network comprised of four kernel layers. In the first and second layers, a stimulus image was preprocessed by the Gabor filter and the local max operation, was applied identically to the candidate. Then in the third layer, the candidate scans over the low-level representation of the stimulus image to obtain a 2-dimensional map measuring the Euclidean distance between the candidate and each sub-region of the stimulus. Finally, the global minimum was pooled from the distance map of the third layer to imitate the position invariance of the IT neurons. This minimum distance was translated to a predicted response by the radial basis function.

Next, we collected a massive number of predicted response vectors generated from each fragment; then, we searched for the fragment whose corresponding response vector was most similar to the columnar response vector. To evaluate the similarity between two vectors, we considered two types of correlation coefficients (global and local). See SI Text for additional details.

## Supplementary Information

Supplementary Information 1.

Supplementary Information 2.

Supplementary Information 3.

Supplementary Information 4.

Supplementary Information 5.

Supplementary Information 6.

Supplementary Information 7.

Supplementary Information 8.

Supplementary Information 9.

Supplementary Information 10.

Supplementary Information 11.

Supplementary Information 12.

Supplementary Information 13.

Supplementary Information 14.

Supplementary Information 15.

## Data Availability

The MATLAB codes central to the research (prediction of neural responses from example fragments, and visualization of the part of stimuli captured by the features) are freely available at https://github.com/YunjunNam0225/FragmentAnalysis_2021-02-11. Auxiliary codes are available from the corresponding author upon request. MATLAB 2008b was used to conduct the analysis.
