# Author Correction: View-tuned and view-invariant face encoding in IT cortex is explained by selected natural image fragments

**DOI:** 10.1038/s41598-021-97066-0

**Published:** 2021-09-01

**Authors:** Yunjun Nam, Takayuki Sato, Go Uchida, Ekaterina Malakhova, Shimon Ullman, Manabu Tanifuji

**Affiliations:** 1 Laboratory for Integrative Neural Systems, RIKEN Center for Brain Science, Wako‑shi, Saitama Japan; 2 Research Promotion Division, Fukushima University, Fukushima, Japan; 3 Lab. Physiology of Vision, Pavlov Institute of Physiology, Saint‑Petersburg, Russia; 4 Department of Computer Science and Applied Mathematics, The Weizmann Institute of Science, Rehovot, Israel; 5 Department of Life Science and Medical Bio‑Science, Faculty of Science and Engineering, Waseda University, Shinjuku, Tokyo Japan

Correction to: *Scientific Reports* https://doi.org/10.1038/s41598-021-86842-7, published online 09 April 2021

The original version of this Article contained errors.

The email address for author Manabu Tanifuji was incorrect. The correct email address for Manabu Tanifuji is mana.tanifuji@gmail.com.

Additionally, Reference 29 was incorrectly given as:

Issa, E. B., Bashivan, P., Kar, K., Schmidt, K. & DiCarlo, J. J. Large-scale, high-resolution comparison of the core visual object recognition behavior of humans, monkeys, and state-of-the-art deep artificial neural networks. *J. Neurosci.* **38**, 7255–7269 (2018).

The correct reference is listed below:

Rajalingham, R. *et al.* Large-scale, high-resolution comparison of the core visual object recognition behavior of humans, monkeys, and state-of-the-art deep artificial neural networks. *J. Neurosci.* **38**, 7255–7269 (2018).

The original Article and accompanying Supplementary Information files have been corrected.

