# Supplementary figures and images for: View-tuned and view-invariant face encoding in IT cortex is explained by selected natural image fragments
Source: Sci Rep. 2021 Apr 9;11:7827. doi: 10.1038/s41598-021-86842-7 (PMC8035202; doi:10.1038/s41598-021-86842-7)

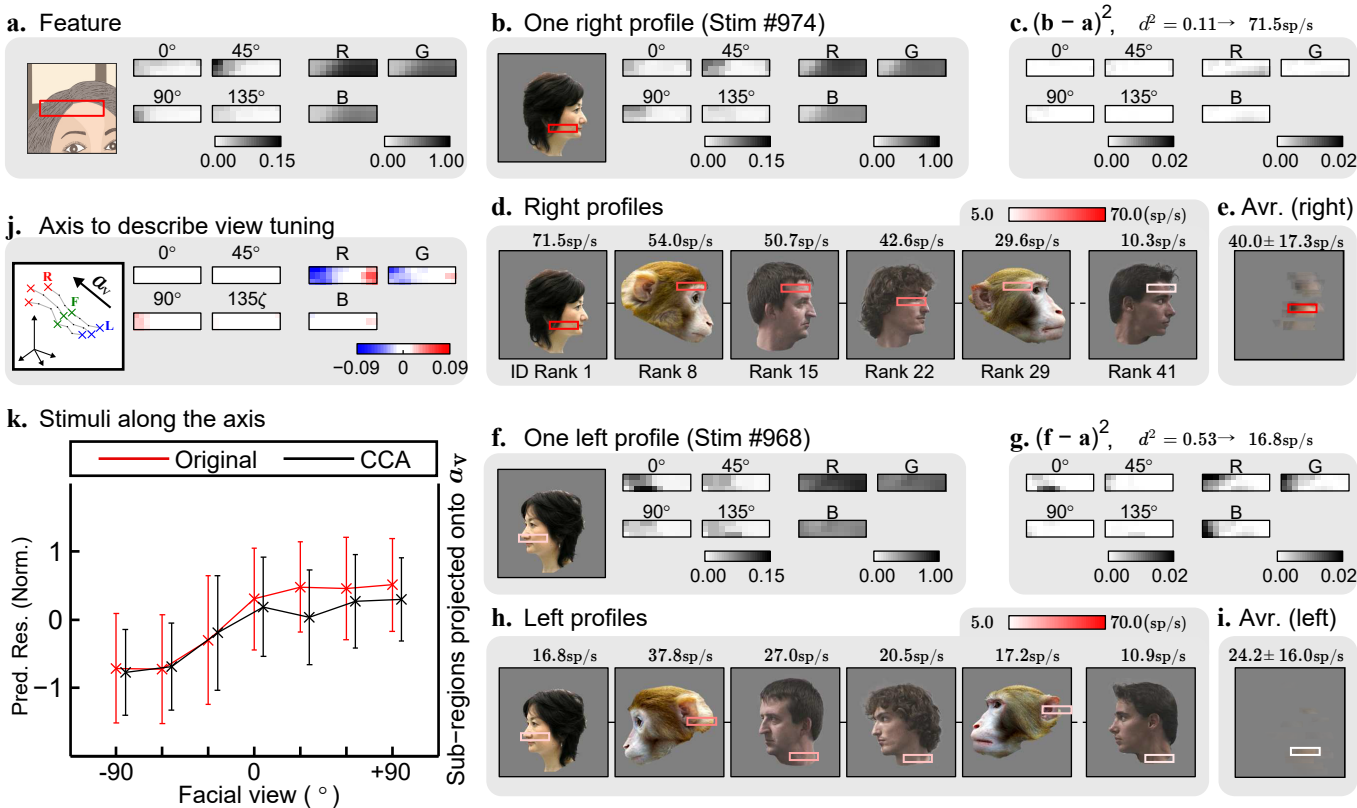

Supplement: Supplementary file 1 — Supplementary Information 1. [file 41598_2021_86842_MOESM1_ESM.pdf]

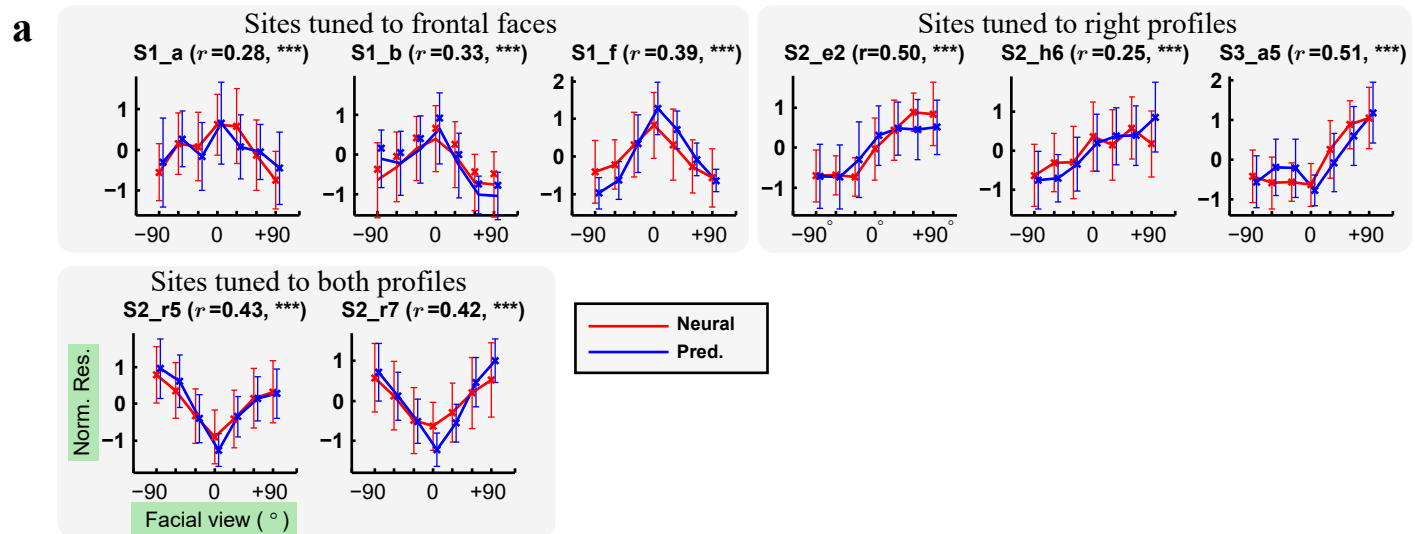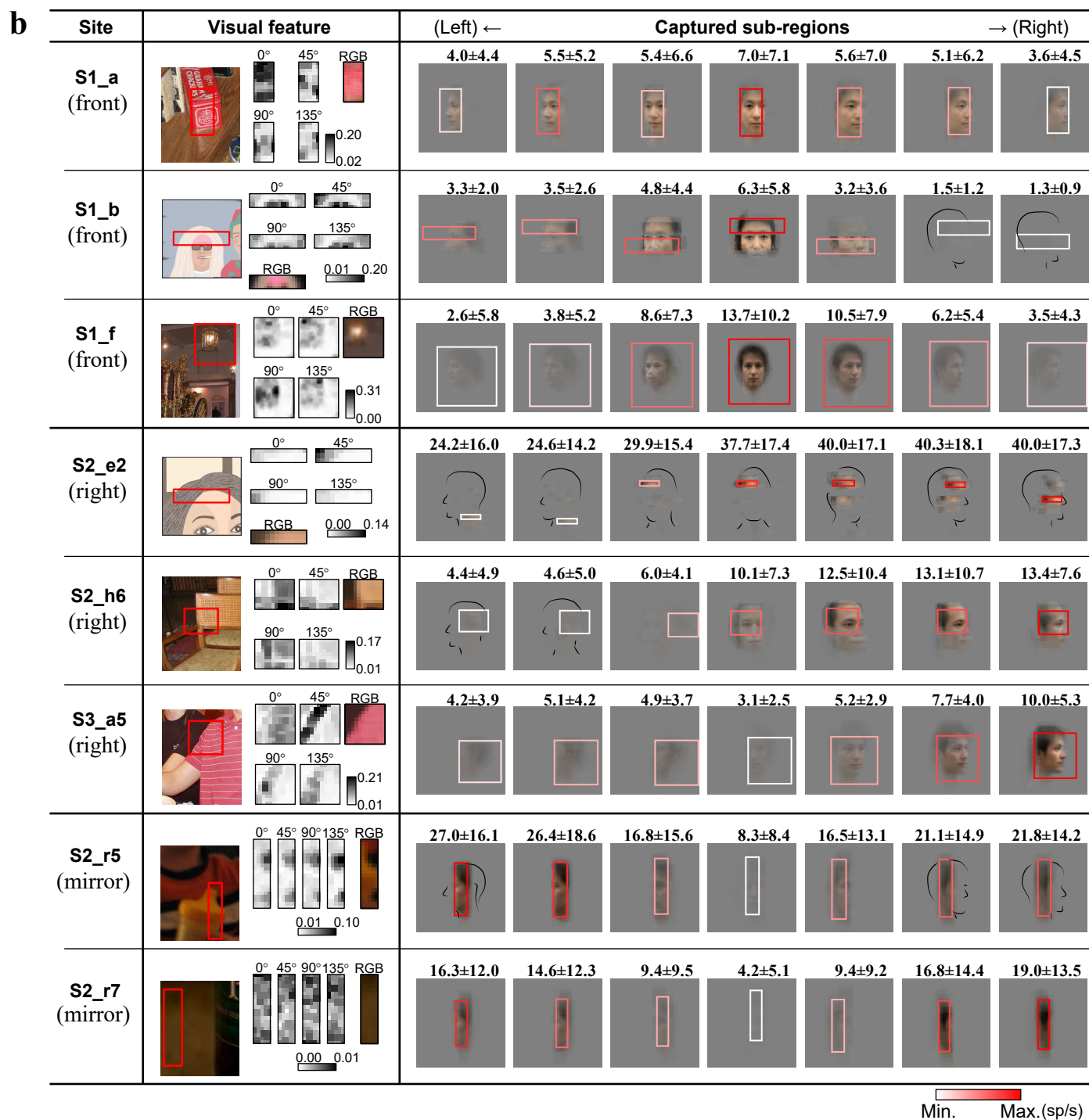

Supplement: Supplementary file 2 — Supplementary Information 2. [file 41598_2021_86842_MOESM2_ESM.pdf]

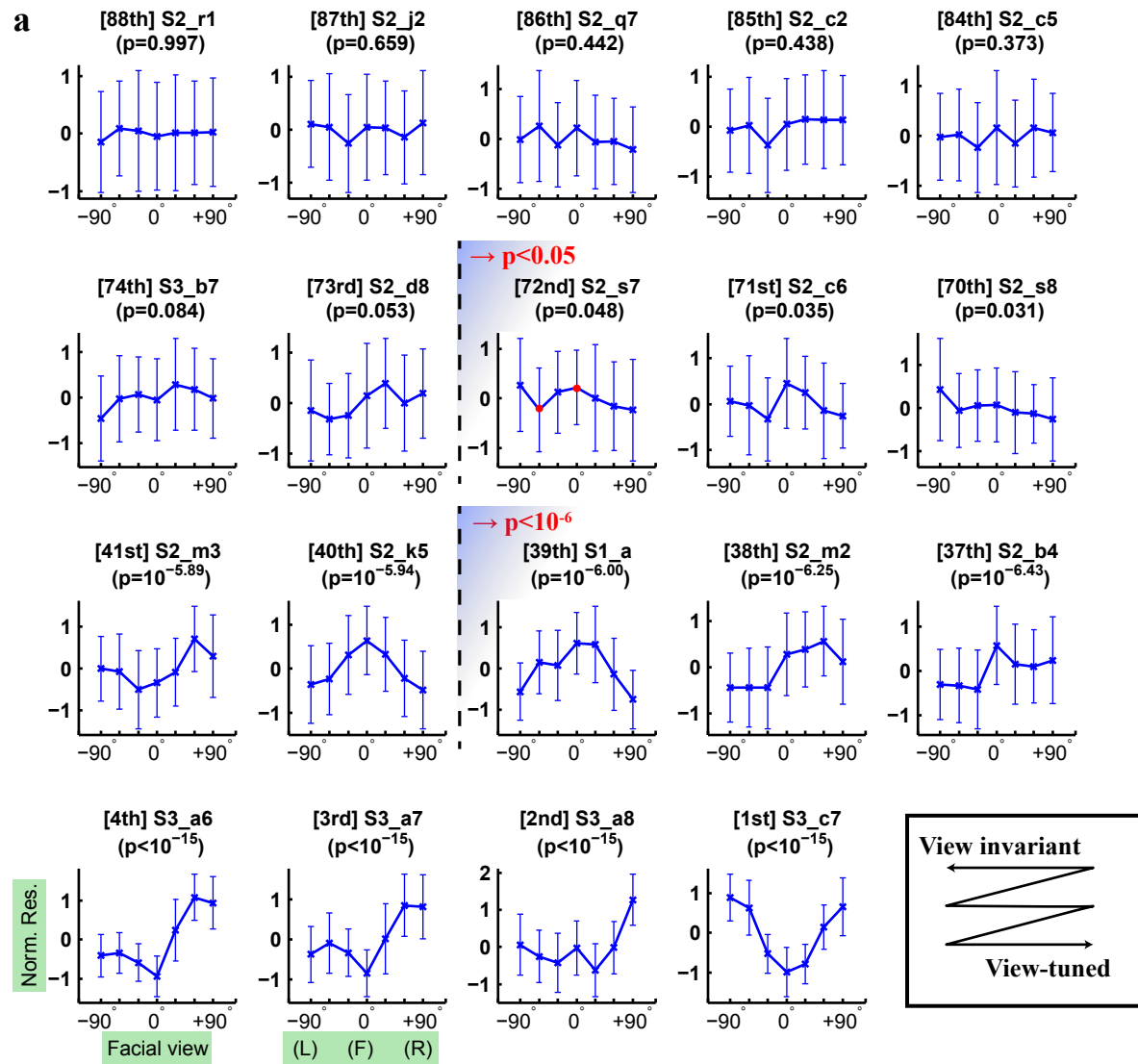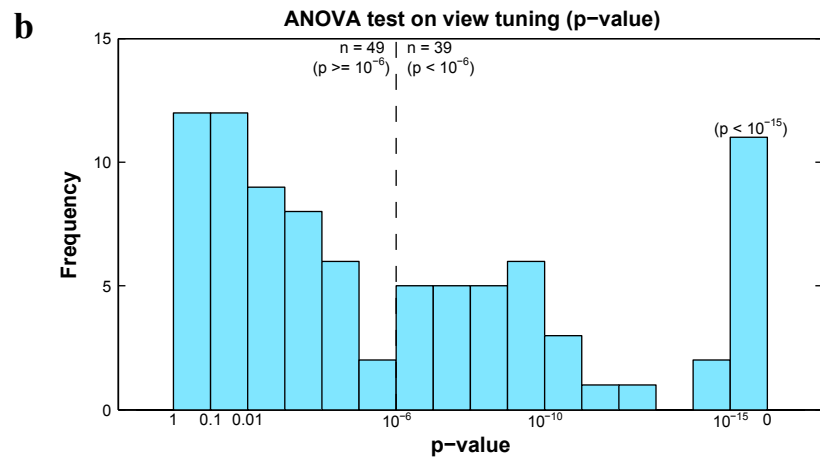

Supplement: Supplementary file 4 — Supplementary Information 4. [file 41598_2021_86842_MOESM4_ESM.pdf]

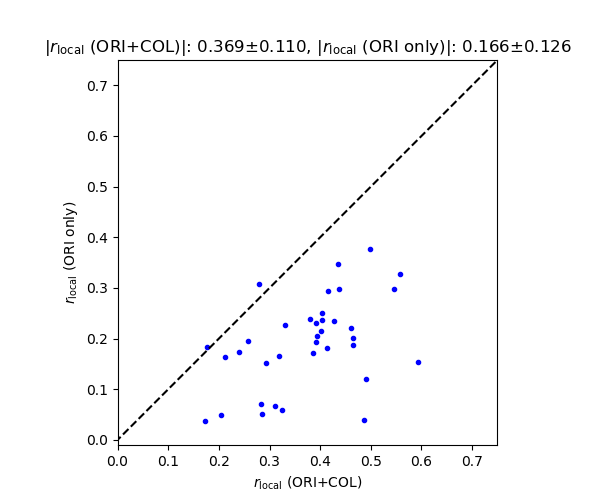

Supplement: Supplementary file 7 — Supplementary Information 7. [file 41598_2021_86842_MOESM7_ESM.png]

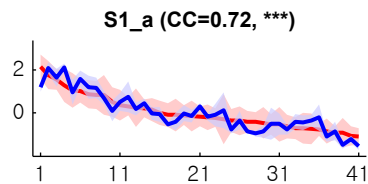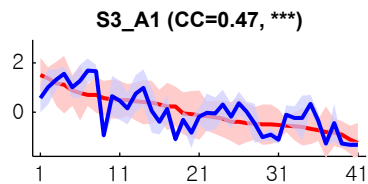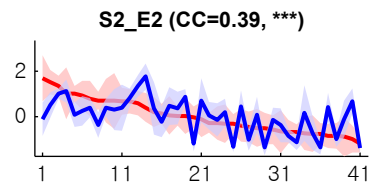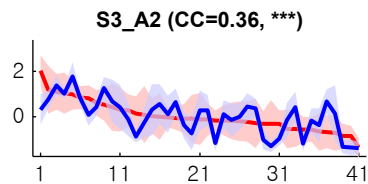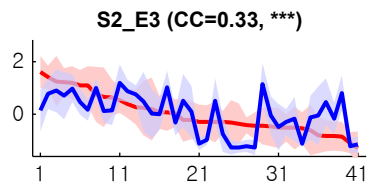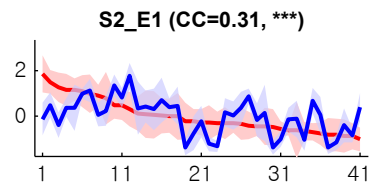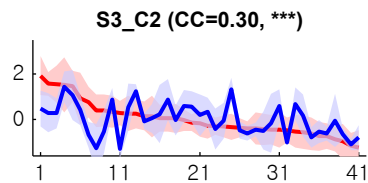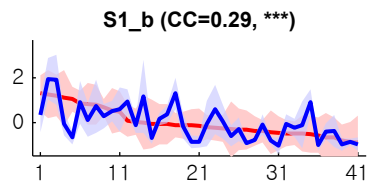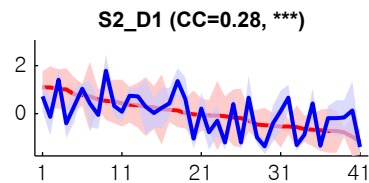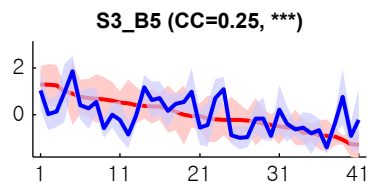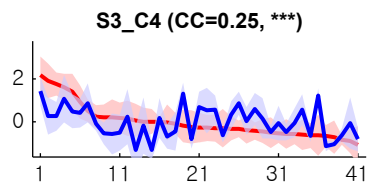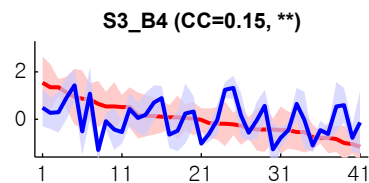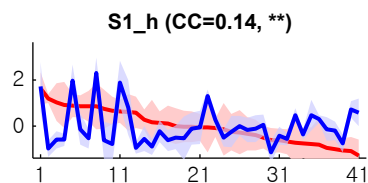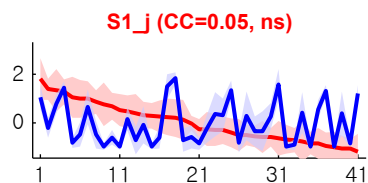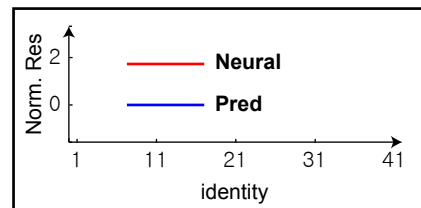

Supplement: Supplementary file 8 — Supplementary Information 8. [file 41598_2021_86842_MOESM8_ESM.pdf]

**a**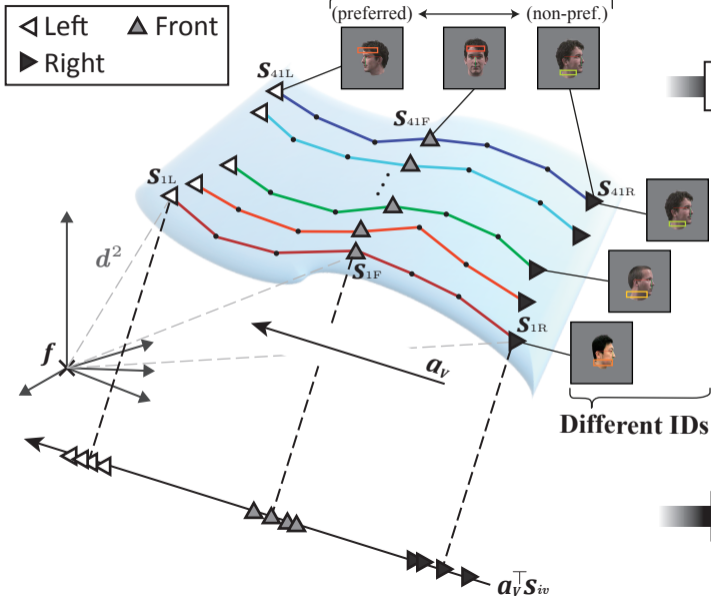**b**

Mean responses for each facial view  $l_v$

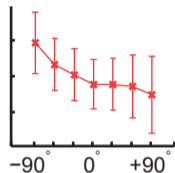**c**

Sub-regions projected onto the axis  $a_v$

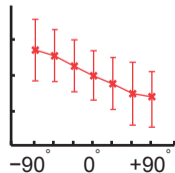

$a_v^\top S$

Supplement: Supplementary file 10 — Supplementary Information 10. [file 41598_2021_86842_MOESM10_ESM.pdf]

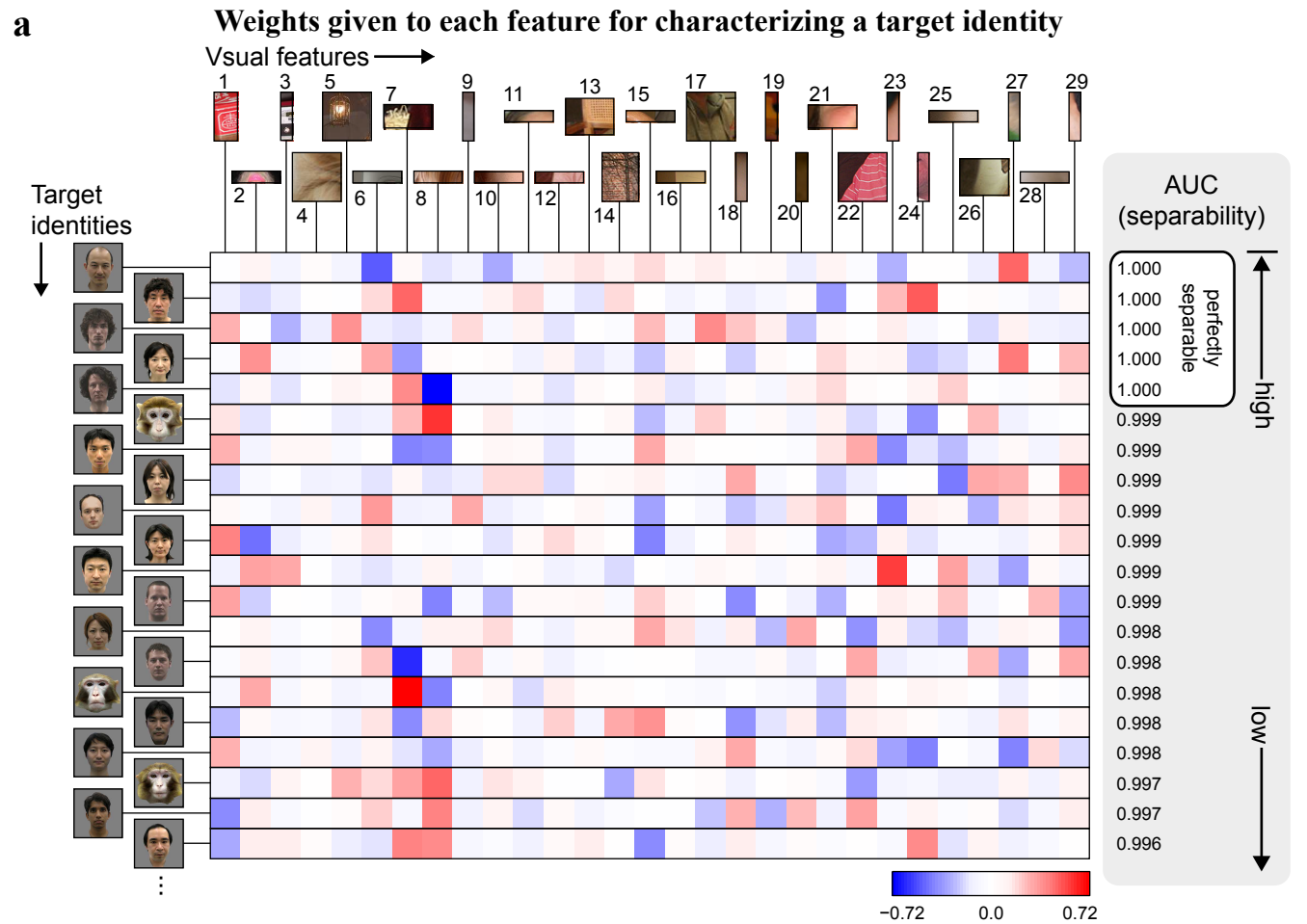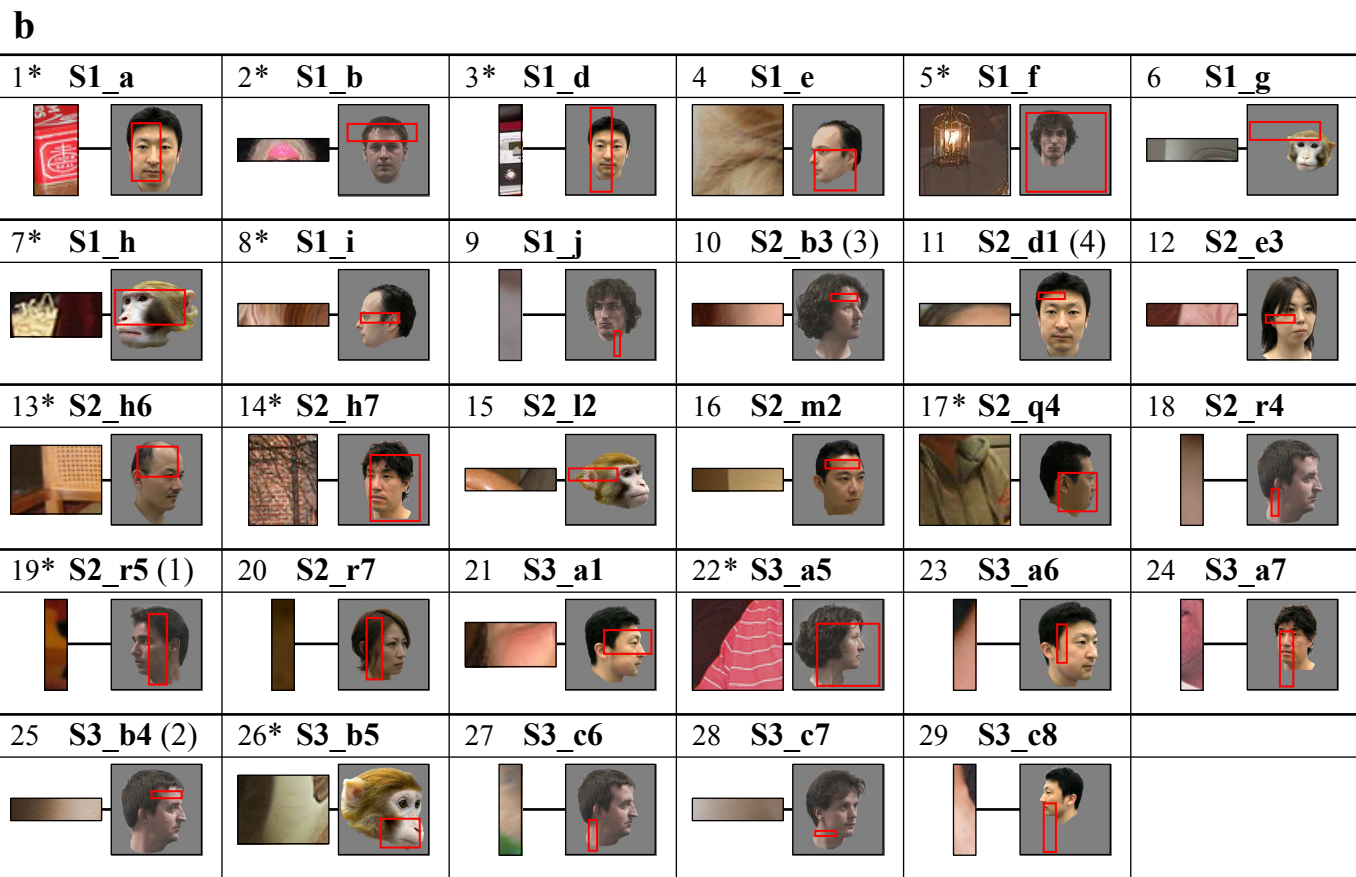

Supplement: Supplementary file 12 — Supplementary Information 12. [file 41598_2021_86842_MOESM12_ESM.pdf]

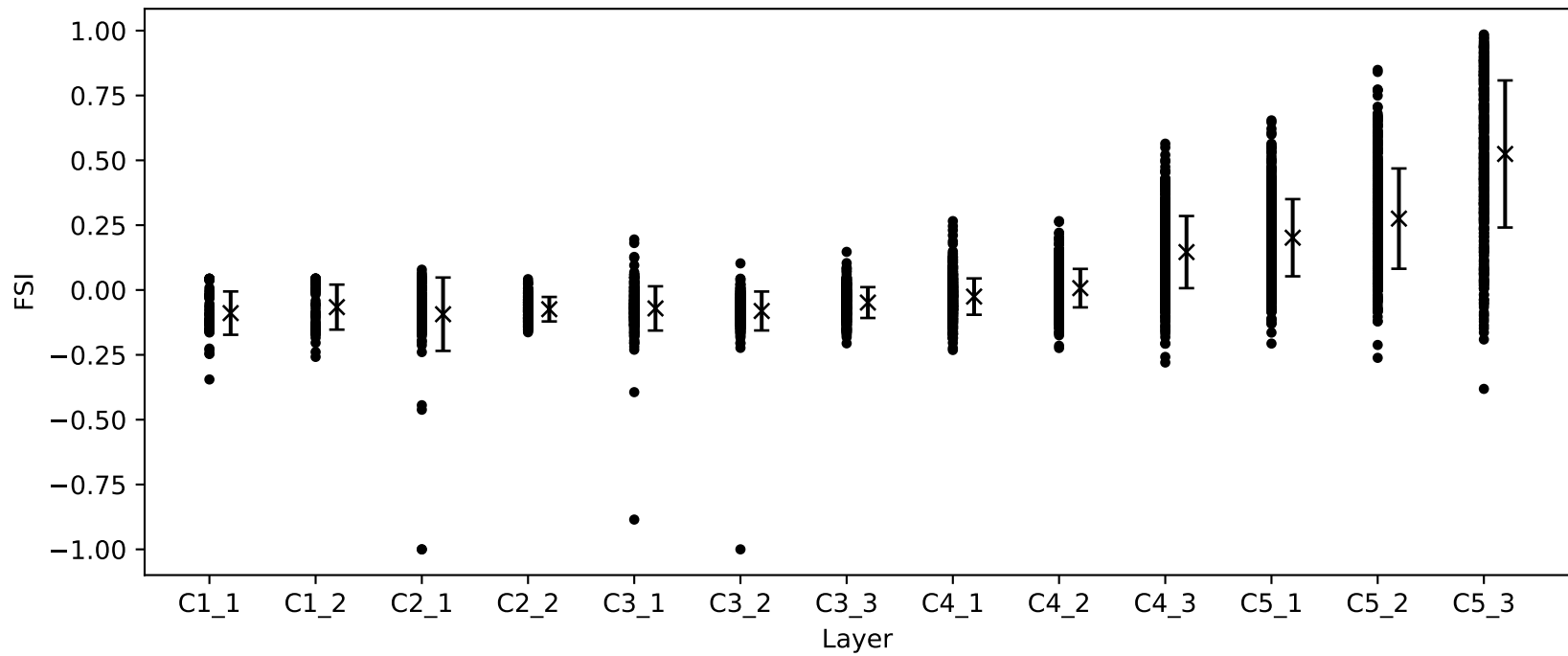

Supplement: Supplementary file 14 — Supplementary Information 14. [file 41598_2021_86842_MOESM14_ESM.pdf]
