# Supplementary material for: View-tuned and view-invariant face encoding in IT cortex is explained by selected natural image fragments
Source: Sci Rep. 2021 Apr 9;11:7827. doi: 10.1038/s41598-021-86842-7 (PMC8035202; doi:10.1038/s41598-021-86842-7)
Supplement: Supplementary file 3 — Supplementary Information 3. [file 41598_2021_86842_MOESM3_ESM.pdf]

Categories		Example images
**View controlled faces (n = 287)**	36 human identities (n = 252)	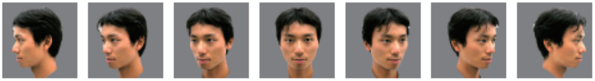 -90° -60° -30° 0° 30° 60° 90° …
	5 monkey identities (n = 35)	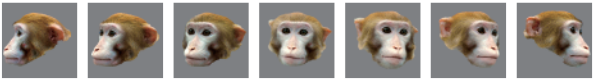 -90° -60° -30° 0° 30° 60° 90° …
**View uncontrolled faces (n = 532)**	Human faces (n = 280)	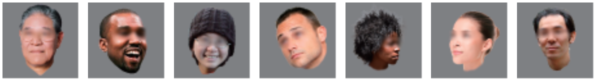 …
	Monkey faces (n = 252)	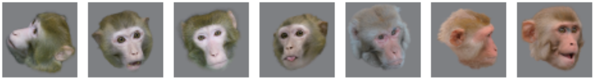 …
**Non-face images (n = 690)**	Primate bodies (n = 100, no head)	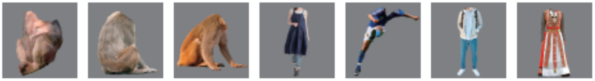 …
	Non-primate animals (n = 200)	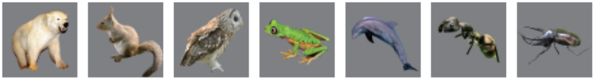 …
	Plants (n = 120)	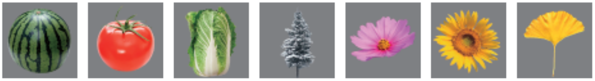 …
	Artficial objects (n = 150)	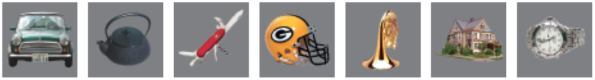 …
	Terrestrial scenes (n = 120)	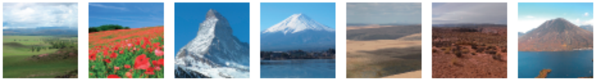 … 10° or 20°
