# Supplementary material for: View-tuned and view-invariant face encoding in IT cortex is explained by selected natural image fragments
Source: Sci Rep. 2021 Apr 9;11:7827. doi: 10.1038/s41598-021-86842-7 (PMC8035202; doi:10.1038/s41598-021-86842-7)
Supplement: Supplementary file 5 — Supplementary Information 5. [file 41598_2021_86842_MOESM5_ESM.pdf]

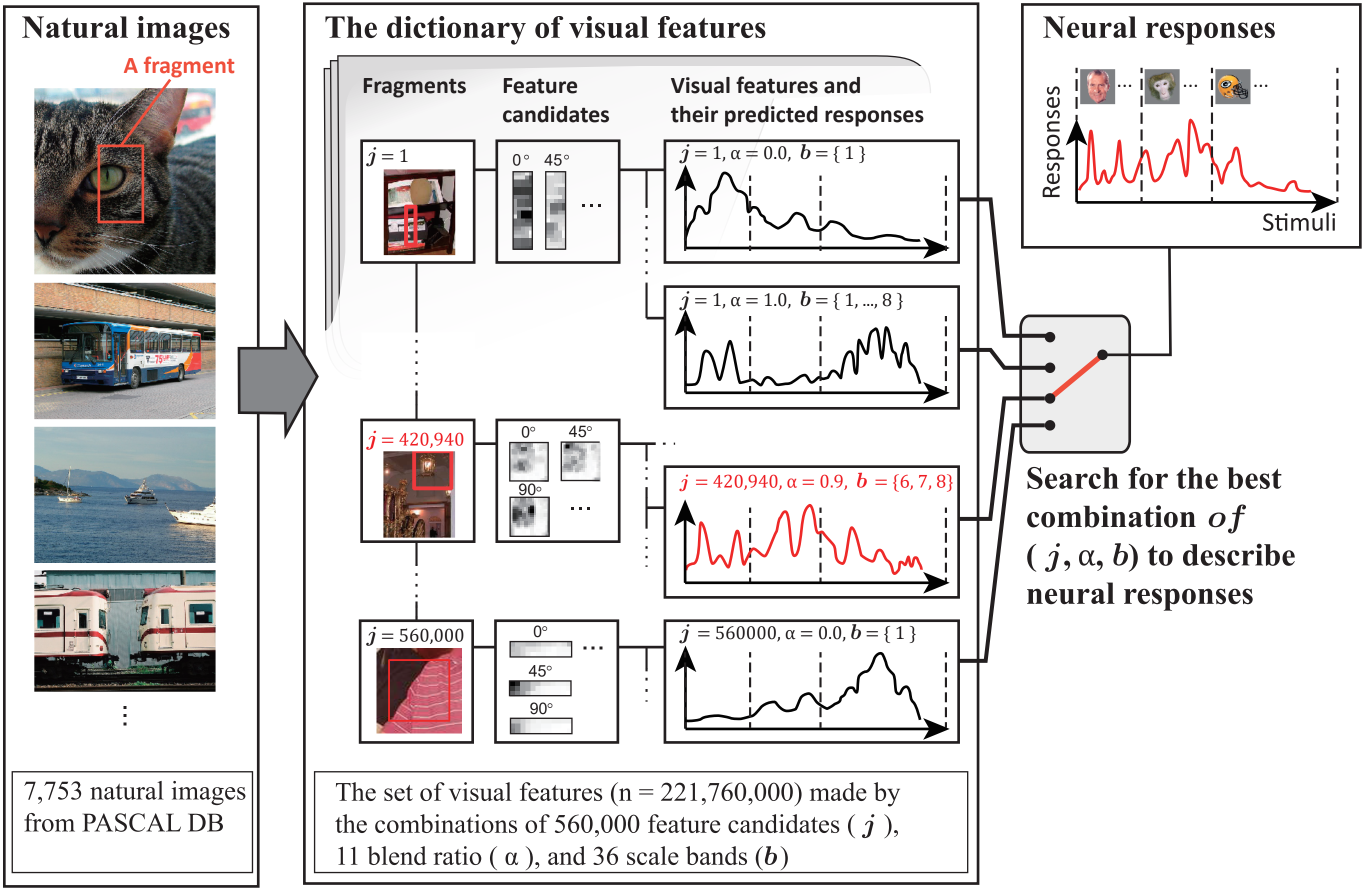

Natural images
A fragment
7,753 natural images from PASCAL DB
The dictionary of visual features
Fragments
Feature candidates
Visual features and their predicted responses
j = 1
0° 45°
j = 1, α = 0.0, b = { 1 }
j = 1, α = 1.0, b = { 1, ..., 8 }
j = 420,940
0° 45° 90°
j = 420,940, α = 0.9, b = {6, 7, 8}
j = 560,000
0° 45° 90°
j = 560000, α = 0.0, b = { 1 }
The set of visual features (n = 221,760,000) made by the combinations of 560,000 feature candidates (j), 11 blend ratio (α), and 36 scale bands (b)
Neural responses
Responses
Stimuli
Search for the best combination of (j, α, b) to describe neural responses
