# Supplementary material for: View-tuned and view-invariant face encoding in IT cortex is explained by selected natural image fragments
Source: Sci Rep. 2021 Apr 9;11:7827. doi: 10.1038/s41598-021-86842-7 (PMC8035202; doi:10.1038/s41598-021-86842-7)
Supplement: Supplementary file 6 — Supplementary Information 6. [file 41598_2021_86842_MOESM6_ESM.pdf]

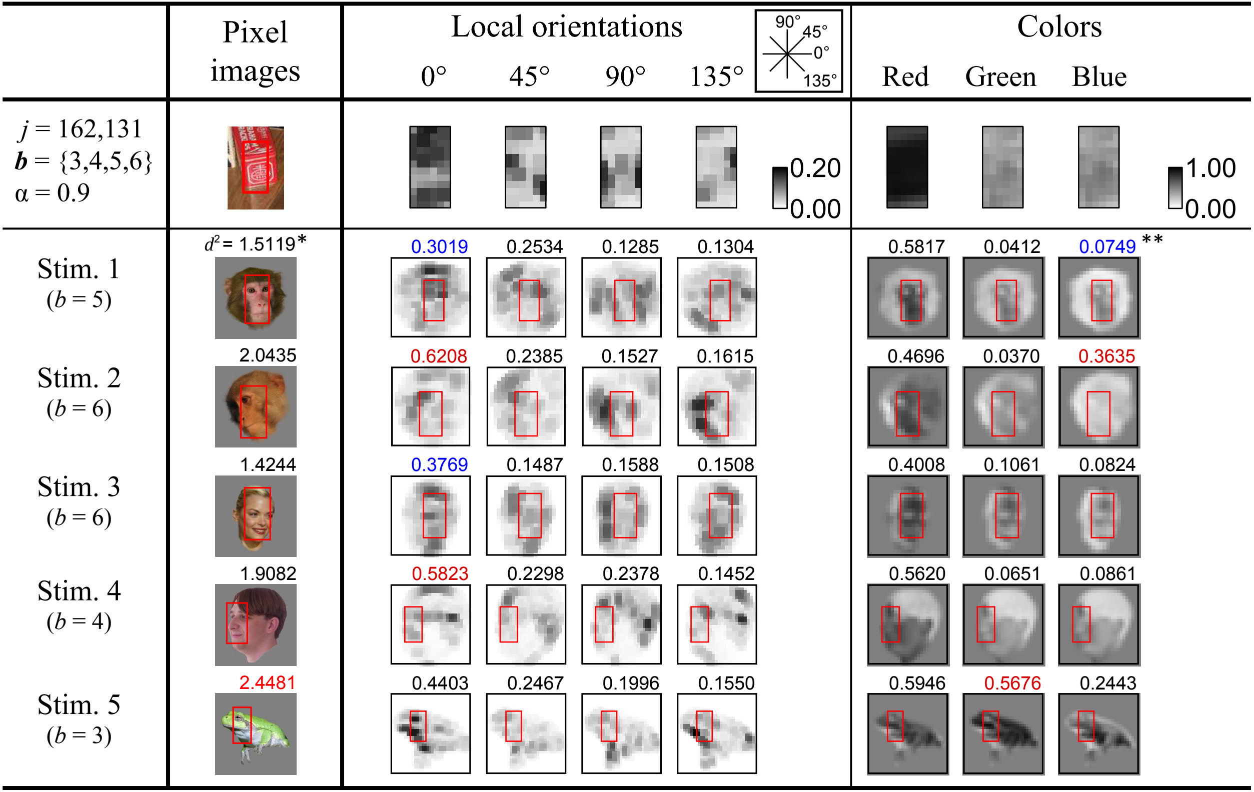

Pixel images
Local orientations
0° 45° 90° 135°
90° 45° 0° 135°
Colors
Red Green Blue
j = 162,131
b = {3,4,5,6}
α = 0.9
0.20
0.00
1.00
0.00
Stim. 1 (b = 5)
d² = 1.5119*
0.3019 0.2534 0.1285 0.1304
0.5817 0.0412 0.0749 **
Stim. 2 (b = 6)
2.0435
0.6208 0.2385 0.1527 0.1615
0.4696 0.0370 0.3635
Stim. 3 (b = 6)
1.4244
0.3769 0.1487 0.1588 0.1508
0.4008 0.1061 0.0824
Stim. 4 (b = 4)
1.9082
0.5823 0.2298 0.2378 0.1452
0.5620 0.0651 0.0861
Stim. 5 (b = 3)
2.4481
0.4403 0.2467 0.1996 0.1550
0.5946 0.5676 0.2443
