# Supplementary material for: View-tuned and view-invariant face encoding in IT cortex is explained by selected natural image fragments
Source: Sci Rep. 2021 Apr 9;11:7827. doi: 10.1038/s41598-021-86842-7 (PMC8035202; doi:10.1038/s41598-021-86842-7)
Supplement: Supplementary file 9 — Supplementary Information 9. [file 41598_2021_86842_MOESM9_ESM.pdf]

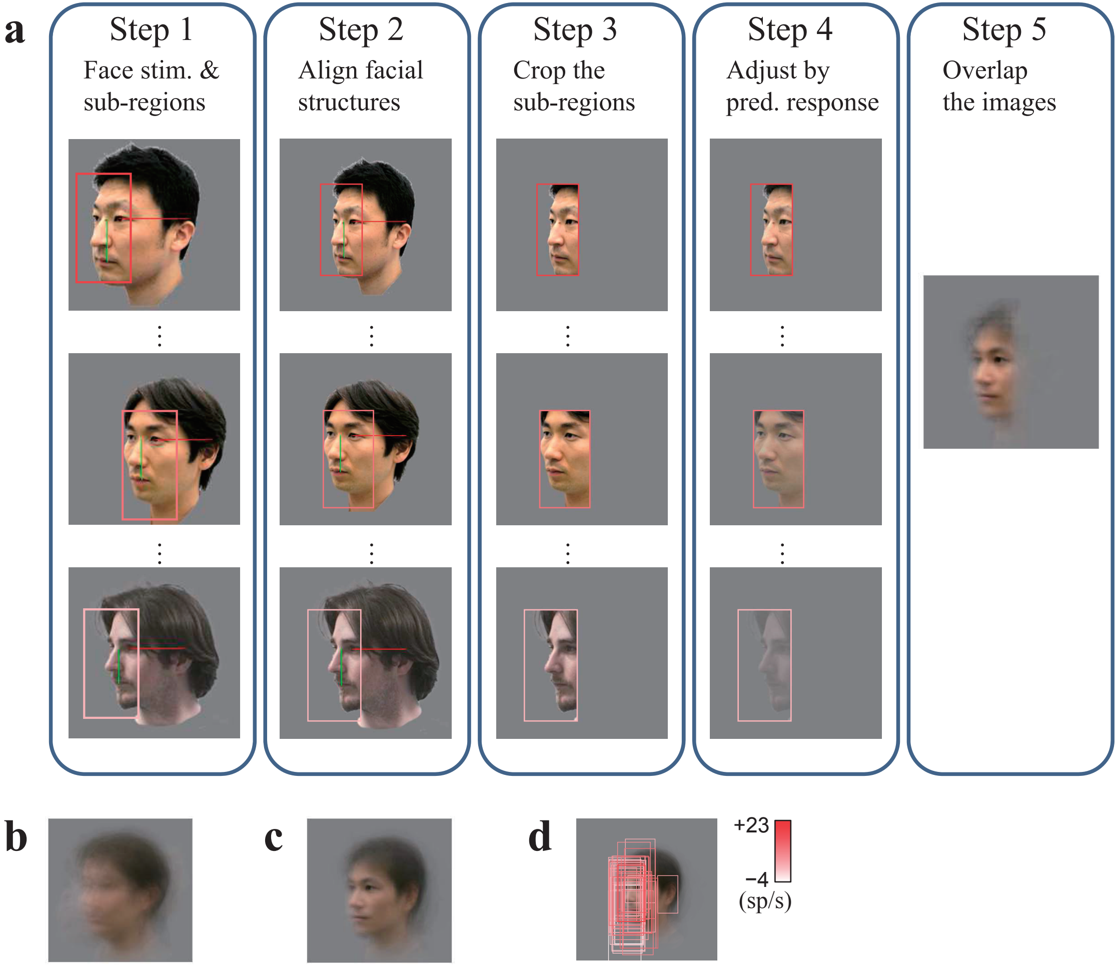
a
Step 1
Face stim. & sub-regions
Step 2
Align facial structures
Step 3
Crop the sub-regions
Step 4
Adjust by pred. response
Step 5
Overlap the images
b
c
d
+23
−4
(sp/s)
