# Supplementary material for: View-tuned and view-invariant face encoding in IT cortex is explained by selected natural image fragments
Source: Sci Rep. 2021 Apr 9;11:7827. doi: 10.1038/s41598-021-86842-7 (PMC8035202; doi:10.1038/s41598-021-86842-7)
Supplement: Supplementary file 11 — Supplementary Information 11. [file 41598_2021_86842_MOESM11_ESM.pdf]

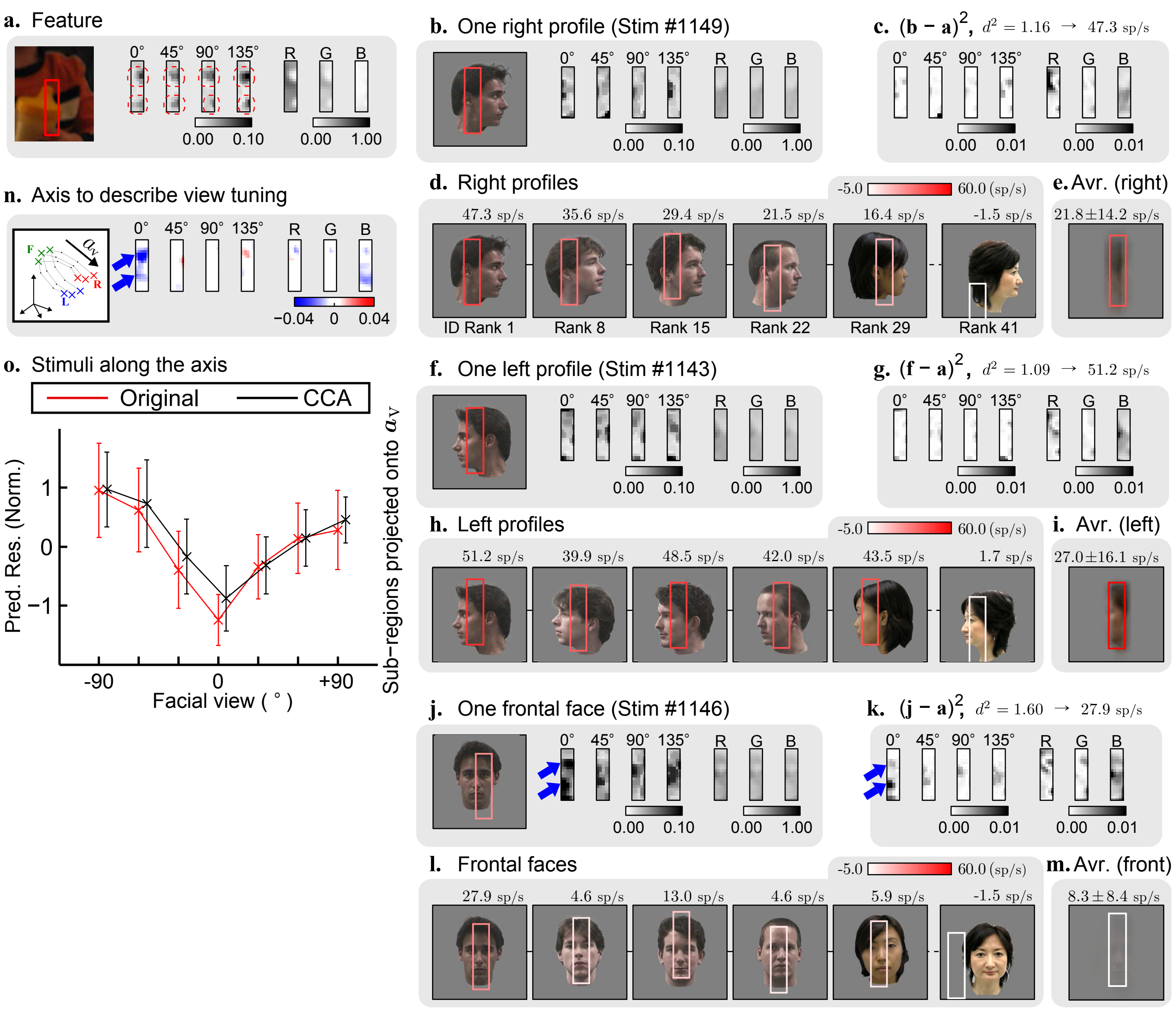

a. Feature
0° 45° 90° 135° R G B
0.00 0.10 0.00 1.00
b. One right profile (Stim #1149)
c. (b − a)^2, d^2 = 1.16 → 47.3 sp/s
0.00 0.01 0.00 0.01
d. Right profiles
-5.0 60.0 (sp/s)
47.3 sp/s 35.6 sp/s 29.4 sp/s 21.5 sp/s 16.4 sp/s -1.5 sp/s
ID Rank 1 Rank 8 Rank 15 Rank 22 Rank 29 Rank 41
e. Avr. (right)
21.8±14.2 sp/s
n. Axis to describe view tuning
F R L a_V
−0.04 0 0.04
o. Stimuli along the axis
Original CCA
Pred. Res. (Norm.)
Facial view (°)
-90 0 +90
Sub-regions projected onto a_V
f. One left profile (Stim #1143)
g. (f − a)^2, d^2 = 1.09 → 51.2 sp/s
h. Left profiles
51.2 sp/s 39.9 sp/s 48.5 sp/s 42.0 sp/s 43.5 sp/s 1.7 sp/s
i. Avr. (left)
27.0±16.1 sp/s
j. One frontal face (Stim #1146)
k. (j − a)^2, d^2 = 1.60 → 27.9 sp/s
l. Frontal faces
27.9 sp/s 4.6 sp/s 13.0 sp/s 4.6 sp/s 5.9 sp/s -1.5 sp/s
m. Avr. (front)
8.3±8.4 sp/s
