# Supplementary material for: View-tuned and view-invariant face encoding in IT cortex is explained by selected natural image fragments
Source: Sci Rep. 2021 Apr 9;11:7827. doi: 10.1038/s41598-021-86842-7 (PMC8035202; doi:10.1038/s41598-021-86842-7)
Supplement: Supplementary file 13 — Supplementary Information 13. [file 41598_2021_86842_MOESM13_ESM.pdf]

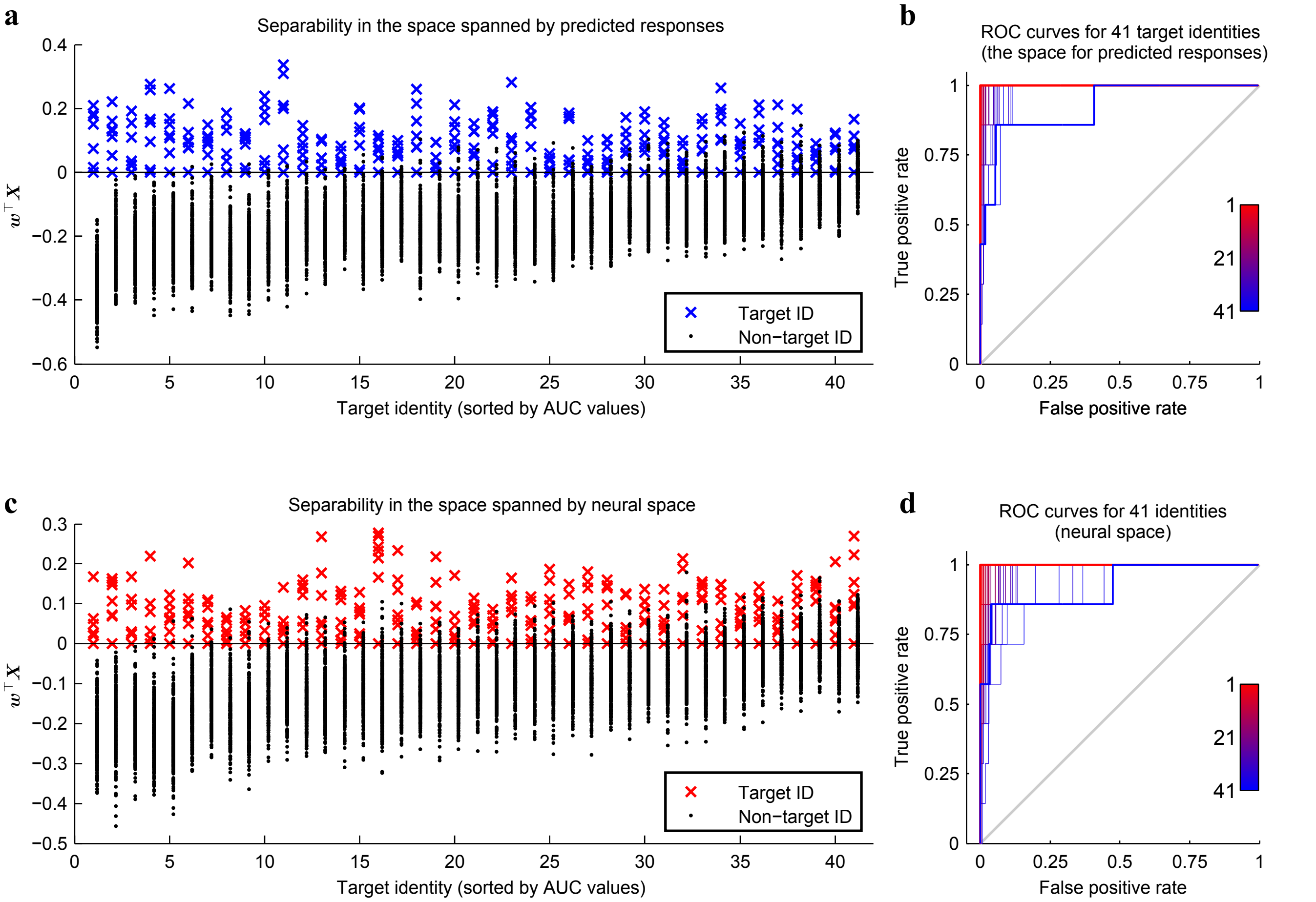

a
Separability in the space spanned by predicted responses
$w^\top X$
Target identity (sorted by AUC values)
Target ID
Non−target ID
b
ROC curves for 41 target identities
(the space for predicted responses)
True positive rate
False positive rate
1
21
41
c
Separability in the space spanned by neural space
$w^\top X$
Target identity (sorted by AUC values)
Target ID
Non−target ID
d
ROC curves for 41 identities
(neural space)
True positive rate
False positive rate
1
21
41
