# Supplementary material for: View-tuned and view-invariant face encoding in IT cortex is explained by selected natural image fragments
Source: Sci Rep. 2021 Apr 9;11:7827. doi: 10.1038/s41598-021-86842-7 (PMC8035202; doi:10.1038/s41598-021-86842-7)
Supplement: Supplementary file 15 — Supplementary Information 15. [file 41598_2021_86842_MOESM15_ESM.docx]

**View-tuned and view-invariant face encoding in IT cortex is explained by selected natural image fragments**

Yunjun Nam^1^, Takayuki Sato^2^, Go Uchida^1^, Ekaterina Malakhova^3^, Shimon Ullman^4^, and Manabu Tanifuji^1^,^5*^

*^1^Laboratory for Integrative Neural Systems, RIKEN Center for Brain Science, Wako-shi, Saitama, Japan*

*^2^Research Promotion Division, Fukushima University, Fukushima, Japan*

*^3^Lab. physiology of vision, Pavlov Institute of Physiology, Saint-Petersburg, Russia*

*^4^Department of Computer Science and Applied Mathematics, The Weizmann Institute of Science, Rehovot, Israel*

*^5^Department of Life Science and Medical Bio-Science, Faculty of Science and Engineering, Waseda University, Shinjuku, Tokyo, Japan.*

* email: mana.tanifuji@gmail.com

* Correspondence should be addressed to Manabu Tanifuji

Supplementary Information Text

**The online links to the figures with the original fragment images.**

For Figs.5, 8, S10, S11, we replaced the human face images into illustrations because of the copy right regulation of Scientific report. The figures with the original images can be downloaded from the links below.

Fig. 5: <https://github.com/YunjunNam0225/FragmentAnalysis_2021-02-11/blob/main/supplementary_figures/Fig5_Fragments_GITHUB.pdf>

Fig. 8: <https://github.com/YunjunNam0225/FragmentAnalysis_2021-02-11/blob/main/supplementary_figures/Fig8_ViewInvariance_GITHUB.pdf>

Fig. S10: <https://github.com/YunjunNam0225/FragmentAnalysis_2021-02-11/blob/main/supplementary_figures/FigS10_ExplainingRightProfileTuning_GITHUB.pdf>

Fig. S11: <https://github.com/YunjunNam0225/FragmentAnalysis_2021-02-11/blob/main/supplementary_figures/FigS11_ViewTuning_AdditionalSites_GITHUB.pdf>

**Visual stimuli.**

We recoded neural responses for 1,509 object images (Fig. S1), consisting of view controlled faces (n = 287), view uncontrolled faces (n = 532), and non-face objects (n = 690). We used the neural responses obtained from the view uncontrolled faces and the non-face objects for identification of the features of columns (feature identification stimulus set). The identified features were then used to predict responses to the view controlled face set, which were compared with the neural responses to the view controlled face set. There was no overlap of images between the view uncontrolled faces and the view controlled faces.

These stimuli were embedded on a gray background and presented to three macaque monkeys (*Macaca fuscata*, S1—female, S2—male, and S3—male), through a 21 inch CRT monitor placed 57 cm in front of their eyes. The size of the objects and faces was set to 20° (for S1 and S3) or 10° (for S2) in a visual angle. Because the monkeys were anesthetized during the recordings, the stimuli were jittered by 0.15° of the visual angle in every 12 ms. Please refer to our previous papers for further details^1,2^.

**Columnar response recording.**

We collected neural data from three macaque monkeys under anesthesia. We placed a titanium recording chamber (inner diameter, 18mm) on the skull just above the dorsal part of the anterior IT cortex (TEad), removed the bone and dura inside the chamber, and then covered it with a glass coverslip to protect the exposed cortex. The chamber was positioned at the center of the anterior, middle temporal sulcus (AP axis), and between the superior temporal sulcus and anterior, middle temporal sulcus (DV axis). The positions of the sulci were identified with an MRI scan conducted beforehand. During the recordings, the glass coverslip was replaced with one with a small hole where we penetrated an electrode perpendicularly to the cortical surface. When we placed the chamber, the monkeys were initially anesthetized with an intramuscular injection of ketamine hydrochloride (5.8 mg/kg), and then deeply anesthetized with an intraperitoneal injection of pentobarbital sodium (35 mg/kg). We maintained deep anesthesia by supplemental intravenous injections of pentobarbital sodium (5–10 mg). Rectal temperature was monitored and maintained at 37.6 °C. During the recordings, the monkeys were artificially ventilated with a mixture of N_2_O, O_2_, and isoflurane (70% N_2_O, 30% O_2_, 0.25%–2.0% isoflurane). We monitored the ECG and EEG continuously and maintained deep anesthesia by adjusting the concentration of isoflurane between 0.25 and 2.0%. Expired CO_2_ concentration and rectal temperature were also monitored and were maintained between 3.5 and 4.5% and at 37.6 °C, respectively. The experimental protocol was approved by the Experimental Animal Committee of the RIKEN Institute and followed the guidelines of the RIKEN Institute and the National Institutes of Health. Please refer to the references for further details^2^.

We recorded neural activities from the columnar regions of the three monkeys. We used a 16 channel Plexon, V-Probe electrode (the distance between adjacent channels = 150 μm) for S1, and an 8 channel NeuroNexus electrode (the distance = 300 μm) for S2 and S3. We penetrated the electrode perpendicularly to the surface of the exposed cortex, and we averaged the spiking activities recorded from the channels positioned within the cortex to obtain a columnar activity readout^2^.

Each of the visual stimuli was presented for 100 ms, and the time interval between the two stimuli was 200 ms. Columnar activities for each stimulus were calculated by subtracting the number of spikes in the baseline period (−50 to 50 ms from the stimulus onset) from that found in the post-stimulus onset period (70 to 220 ms from the stimulus onset). During the recording from each site, each object was presented 12 times, and the neural response for the object was calculated by averaging columnar activities for these 12 trials. The sequence of 18,108 stimuli (= 1,509 images × 12 trials) were randomly shuffled to avoid repetition suppression.

The responses were recorded from 190 sites in total from the three monkeys, S1 (n = 33), S2 (n = 133), and S3 (n = 24). Among the 190 sites, we deemed 152 sites as selective to face images against non-face images (face selectivity index > 1/3) ^3^. We calculated the average responses for 819 face stimuli $(=\left| \boldsymbol{y}_{\text{F}} \right|)$ and 690 non-face stimuli $(=\left| \boldsymbol{y}_{\text{NF}} \right|)$, then measured the face selectivity index ($FSI$) as follows,

$\text{ }\text{FSI}\text{ }\mathbf{=}\frac{\left| \mathbf{y}_{\text{F}} \right|\mathbf{-}\left| \mathbf{y}_{\text{NF}} \right|}{\left| \mathbf{y}_{\text{F}} \right|\mathbf{+}\left| \mathbf{y}_{\text{NF}} \right|}$. (1)

We further selected 88 reliable sites by measuring the repeatability in their neural responses defined by the correlation between the object responses that averaged over odd trials and those averaged over even trials with the Spearman-Brown correction^4^. We rejected 64 sites whose repeatability was smaller than 0.5, in which responses evoked by stimuli explained less than 25.0% of the total variation (see Fig. 4 for the prediction performance against the repeatability).

**Drawing view tuning curves of the sites tuned to particular facial views.**

From the 88 recording sites deemed to be reliable and face-selective, view tuning curves were drawn from their responses to the view-controlled face stimuli. The view-controlled face stimuli consisted of 287 face images taken from seven facial views—from the left ($L$) to the right ($R$) profiles taken every 30° for the 41 identities (36 humans and 5 monkeys). Let $v\in\left\{ L, \cdots, R \right\}$ and $i\in\left\{ 1, \cdots,41 \right\}$ be the labels for the views and identities, respectively. Then, the neural response to a single stimulus was provided by $y_{iv}$. To discard response variations across identities, we calculated a standard score (or z-score) $z_{iv}$, as follows,

$\text{z}_{\text{iv}}=\frac{\text{y}_{\text{iv}}-\text{μ}_{\text{i}}}{\text{σ}_{\text{i}}}$ (2)

Please note that $\mu_{i}$ and $\sigma_{i}$ are the mean and standard deviation of the seven responses $([y_{iL}, \ldots, y_{iR}])$ from the $i$-th identity. After calculating the z-scores, the mean of $z_{iv}$ for each view was averaged across identities, then plotted as the view tuning curve (Fig. 1 and Fig. S2A). The standard deviations in each view were also visualized via error bars to represent the variations across identities.

By examining the view tuning curves from the 88 sites, 39 sites were selected whose responses were tuned to particular facial views. In order to quantify the specificity of the neural responses to the particular facial views, we utilized the ANOVA test, where the NULL hypothesis was “the mean responses for seven views are identical.” Since the p-value from this test was defined as the probability of observing a curve given that the NULL hypothesis was true (mean values for the seven views were the same; thus, the view tuning curve was completely flat), it can be used as a measure to evaluate the specificity. In Fig. S2A, view tuning curves, were visualized with their p-values sorted in descending order; with the decrease of the p-value, the tuning curves become more specific to a particular facial view (or multiple “views” in the case of the mirror symmetric view tuning preferring both profiles, such as S3_c7), with smaller variations across identities. This result showed that the p-value from the ANOVA test could be utilized as a measure to quantify the mentioned specificity.

Next, we attempted to find a proper threshold to determine whether the responses from a site were view-tuned or view invariant. In statistical hypothesis testing, 0.05 was used conventionally as a cutoff level to reject the null hypothesis. However, from the sites whose p-values were close to 0.05 (for example, site S2_s7 in the second row of Fig. S2A), the difference across the views was not significant enough (p > 0.05) even in a comparison between the most (0° in the case of S2_s7, red dot) and least (−60°, red dot) preferred views. To find a more restrictive threshold, we inspected the curves exhibiting a reduced threshold, and arbitrarily selected 10^-6^ as the threshold to select the view-tuned sites. In the sites whose p-values were smaller than this threshold (see the third row of Fig. S2A), their tuning curves were specific enough to be expressed in a simple term, such as “tuned to frontal faces” (S1_a, S2_b4), “tuned to right profiles” (S2_m2, S3_a6), and “tuned to both profiles” (S3_c7). With this threshold, 39 of the 88 sites (= 44.3%) were categorized as view-tuned sites (see Fig. S2B for the histogram of the p-values from each site).

**Fragment-based analysis to search for optimal visual features.**

Next, we identified the optimal feature descriptions for the thirty-nine view tuned sites by using the fragment-based approach proposed by Owaki et al^5^. This approach searched for the optimal visual features form the database (VOC 2010^6^) of natural image fragments (the left side of Fig. S3). These images were cut into 560,000 fragments (the center of Fig. S3) having various shapes (the height-to-width ratio was varied from 1:0.25, 1:0.5, 1:0.75, 1:1, 0.75:1, 0.5:1, to 0.25:1) and sizes (among width and height, a longer side has one of the four pixel lengths: 34, 50, 66, or 82). To emulate low-level visual processing prior to the visual information reaching the IT cortex, we utilized the Gabor function and local max operations to preprocess a fragment image in terms of the orientation and color (see band 1 in Table S1^7^ for the parameters used).

To preprocess the images with respect to their orientation, the images were converted to grayscale, then convolved using the Gabor function with four different orientations ($o\in\left\{ 0^{\circ}, 45^{\circ}, 90^{\circ}, 135^{\circ} \right\}$). These convolved images were respectively fed to a local max operation, then transformed into four images with the same width $(W)$ and height $\left( H \right),$ denoted by $\boldsymbol{F}_{\boldsymbol{o}}\boldsymbol{\in}\mathbb{R}^{W\times H\times4}$ (see the third column of Fig. S4 for examples). To preprocess the images with respect to color, we applied the same local max operation to the three color channels ($c\in\left\{ R, G, B \right\}$), then obtained three preprocessed images, denoted by $\boldsymbol{F}_{\boldsymbol{c}}\boldsymbol{\in}\mathbb{R}^{W\times H\times3}$ (the fourth column of Fig. S4). By concatenating $\boldsymbol{F}_{\boldsymbol{o}}$ and $\boldsymbol{F}_{\boldsymbol{c}}$, a single feature candidate $\boldsymbol{F}_{\{\boldsymbol{o},\boldsymbol{c}\}}\boldsymbol{\in}\mathbb{R}^{W\times H\times7}$ (see the center of Fig. S3; see Fig. S4 for examples) was obtained. In this study, each of the seven images was referred to as a *channel*, and each value in the seven channels will be referred to as a *component*.

After producing a massive number (n = 560,000) of feature candidates from the image database, each candidate was utilized to generate a response vector, where each vector element represented the predicted responses to one of the stimuli (the center of Fig. S3). The Cortical Network Simulator was used for the calculation of the responses^8^ . For each pair of a fragment and stimulus, the predicted response was calculated in the following way. First, a stimulus image was preprocessed by the Gabor filter and local max operation similarly to the natural image fragments. Eight different parameter settings^7^ (Table S1) were prepared to consider the scale invariance of the IT columns^9^. The first setting, coded as band 1 $(b=1)$, exhibited small sizes in the Gabor filter (7 × 7) and local max window (8 × 8); thus, each component of the preprocessed image encoded a small part (14 × 14 pixels) of the stimulus image. The area encoded by the single component gradually increased in the higher bands, on the last band $(b=8)$, the area covered by the single component expanded to 56 × 56, encompassing almost a quarter of a stimulus image.

For a given band $b$, a stimulus image was converted into preprocessed images in seven channels (see the five stimuli in Fig. S4). Then, by utilizing a feature candidate $\boldsymbol{F}_{\{\boldsymbol{o},\boldsymbol{c}\}}$ as the template, we scanned over the preprocessed stimulus image to locate the sub-region which best matched the candidate. This took into account the position invariance of the IT neurons. Let us assume one sub-region of the preprocessed stimulus image ($\boldsymbol{S}_{\{\boldsymbol{o},\boldsymbol{c}\}}\boldsymbol{\in}\mathbb{R}^{H\times W\times7}$), which had the same height ($H$) and width ($W$) as the candidate $\boldsymbol{F}_{\{\boldsymbol{o},\boldsymbol{c}\}}\boldsymbol{\in}\mathbb{R}^{H\times W\times7}$. With denoting its local orientation channels by $\boldsymbol{S}_{\boldsymbol{o}}\boldsymbol{\in}\mathbb{R}^{H\times W\times4}$ and color channels by $\boldsymbol{S}_{\boldsymbol{c}}\boldsymbol{\in}\mathbb{R}^{H\times W\times3}$, we could calculate the squared Euclidean distance ($d^{2}$) between $\boldsymbol{F}_{\{\boldsymbol{o},\boldsymbol{c}\}}$ and $\boldsymbol{S}_{\{\boldsymbol{o},\boldsymbol{c}\}}$ by

$\text{d}^{\text{2}}\mathbf{=}\text{α}\sum_{\boldsymbol{o}} \left\| \boldsymbol{f}_{\boldsymbol{o}}\boldsymbol{-}\boldsymbol{s}_{\boldsymbol{o}} \right\|^{\boldsymbol{2}}\boldsymbol{+}\text{(1}\boldsymbol{-}\text{α}\text{)}\sum_{\boldsymbol{c}} \left\| \boldsymbol{f}_{\boldsymbol{c}}\boldsymbol{-}\boldsymbol{s}_{\boldsymbol{c}} \right\|^{\boldsymbol{2}}$ (3)

where $\boldsymbol{f}_{o}$, $\boldsymbol{f}_{s}$, $\boldsymbol{s}_{o}$, and $\boldsymbol{s}_{c}$ are the vectorized forms of $\boldsymbol{F}_{o}$, $\boldsymbol{F}_{c}$, $\boldsymbol{S}_{o}$, and $\boldsymbol{S}_{c}$, respectively. The $\alpha$ is the relative weight on the orientation and color channels (simply, *the blend ratio*) that takes one of 11 possible values $\left( \alpha\in\left\{ 0.0, 0.1, \ldots, 1.0 \right\} \right)$. The higher $\alpha$ value implied that the measured distance was more dependent on the local orientations than their colors. After calculating the Euclidean distances between the candidate ($\boldsymbol{F}_{\{\boldsymbol{o},\boldsymbol{c}\}}$) and every existing sub-region ($\boldsymbol{S}_{\{\boldsymbol{o},\boldsymbol{c}\}}$), we located the sub-region with the minimum Euclidean distance (see the red rectangles in Fig. S4). This minimum distance or position invariance will be denoted by $d'$.

The size of the sub-region to be adjusted was enabled to account for scale invariance. Since the size was determined by the band parameter $b$, we searched for the minimum distance by modulating this parameter within the range of $b_{\min}$ and $b_{\max}$, as follows,

$\text{d}\text{''} \left( \text{α, }\text{b} \right)\mathbf{=}\min\left( \left[ \text{d}\text{'} \left( \text{α,}\text{ b}_{\text{min}} \right)\boldsymbol{,\ldots,}\text{d}\text{'} \left( \text{α,}\text{ b}_{\text{max}} \right) \right] \right)$. (4)

In the equation, $d' \left( \alpha,b \right)$ was defined by $d'$ under two given parameters $\alpha$ (the blend ratio) and $b$ (the band), and $d'' \left( \alpha,\boldsymbol{b} \right)$ is the new distance measure considering both position and scale invariance. In the same equation, $\boldsymbol{b}$ denoted one set of consecutive integers from $b_{\min}$ to $b_{\max}$. Since we had eight different bands, the modulation of $b_{\min}$ and $b_{\max}$ could provide 36 different types of $\boldsymbol{b}$ that could be collected as the set $\boldsymbol{B}\boldsymbol{\in}\left\{ \left\{ 1 \right\}, \left\{ 1,2 \right\}, \ldots, \left\{ 1,\ldots,8 \right\}, \left\{ 2 \right\}, \left\{ 2, 3 \right\}, \ldots, \left\{ 2,\ldots,8 \right\} , \ldots, \left\{ 7 \right\}, \left\{ 7,8 \right\}, \left\{ 8 \right\} \right\}$. The visual feature in Fig. S4 has $\boldsymbol{b=}\left\{ 3, 4, 5, 6 \right\}$; therefore, it used one of the four values to predict each of the responses to the five stimuli.

Then, the value of the minimum distance $d'' \left( \alpha,\boldsymbol{b} \right)$ was transferred to the predicted response ($x$) by the radial basis function as follows,

$\text{ }\text{x}\mathbf{=}\exp\left( {\mathbf{-}\left\{ \text{d}\text{''} \left( \text{α, }\text{b} \right) \right\}}^{\mathbf{2}} \right)\mathbf{.}$ (5)

With this function, a good match between the candidate and sub-region was translated to a higher magnitude of the predicted response, and vice versa.

From the equation (5), let us define $x \left( i | j, \alpha,\boldsymbol{b} \right)$ as the predicted response for $i$–th as the stimulus for three given parameters: $j$ (denoting $j$–th feature candidate), $\alpha$ (the blend ratio), and $\boldsymbol{b}$ (the band range). Our next objective was to search for the best combination of the three parameters ($j, \alpha, \text{and} \boldsymbol{b}$), which makes the predicted response vector $\boldsymbol{x}_{(j, \alpha,\boldsymbol{b})}\boldsymbol{\in}\mathbb{R}^{N}$ maximally similar to the actual neural response vector $\boldsymbol{y}\boldsymbol{\in}\mathbb{R}^{N}$, where

$\boldsymbol{x}_{\text{(}\text{j, α, }\text{b}\text{)}}\mathbf{=}\left[ \text{x} \left( \text{1 }\text{|}\text{ j, α, }\text{b} \right)\boldsymbol{, \ldots,}\text{x} \left( \text{N }\text{|}\text{ j, α, }\text{b} \right) \right]$, (6)

and $N$ is the number of views uncontrolled and non-face images (= 1,222).

To achieve this goal, we collected $\boldsymbol{x}_{\left( j, \alpha,\boldsymbol{b} \right)}$ for all of the possible combinations of $j$, $\alpha$, and $\boldsymbol{b}$ (the number of possible combinations was 560,000 ×11 × 36 = 221,760,000; see the center of Fig. S3), then two types of correlations were calculated with the neural response vector $\boldsymbol{y}$. The global correlation $(r_{\text{global}})$ was defined by $\mathrm{corr}\left( \boldsymbol{y},\boldsymbol{x}_{\left( j, \alpha,\boldsymbol{b} \right)} \right)$, where $\mathrm{corr}\left( \right)$ is the function of calculating Pearson’s correlation coefficient between the two vectors with 100 iterations of the delete-half jackknife resampling. The delete-half jackknife resampling was utilized to avoid the identified features being biased to fit a few neural responses among $\boldsymbol{y}$, which has extremely high values due to the experimental noise.

On the other hand, the local correlation $(r_{\text{local}})$ was calculated by $\mathrm{corr}\left( \boldsymbol{y}_{F}\boldsymbol{,}\boldsymbol{x}_{F\left( j, \alpha,\boldsymbol{b} \right)} \right),$ where $\boldsymbol{y}_{\boldsymbol{F}}\boldsymbol{\in}\mathbb{R}^{532}$ and $\boldsymbol{x}_{\boldsymbol{F}\left( j, \alpha,\boldsymbol{b} \right)}\boldsymbol{\in}\mathbb{R}^{532}$ are the subset of, $\boldsymbol{y}$ and $\boldsymbol{x}_{\left( j, \alpha,\boldsymbol{b} \right)}$ that corresponded only to the view of the uncontrolled face stimuli. Finally, we searched for a particular combination of $j$, $\alpha$, and $\boldsymbol{b}$, which satisfies

$\left( \tilde{\text{j}}\text{, }\tilde{\text{ α}}\text{,}\tilde{\text{ }\text{b}} \right)\boldsymbol{=}\underset{\text{j, α, }\text{b}}{\text{arg max}} \left( \text{corr}\left( \boldsymbol{y}\boldsymbol{,}\boldsymbol{x}_{\boldsymbol{(}\text{j, α ,}\text{b}\boldsymbol{)}} \right) \right)$, subject to $\text{corr}\left( \boldsymbol{y}_{\mathbf{F}}\boldsymbol{,}\boldsymbol{x}_{\mathbf{F}\left( \text{j, α, }\text{b} \right)} \right)\boldsymbol{>0.2661}$. (7)

The threshold on $r_{\text{local}}$ (= 0.2661) is the minimum correlation coefficient to reject the null hypothesis that two vectors came from populations with zero correlation at a significance level of 0.05 with the Bonferroni correction. In this study, we will refer to the favorable combination of $\tilde{j}, \tilde{\alpha}$, and $\tilde{\boldsymbol{b}}$ as the *visual feature* for the site.

**Fitting predicted responses to neural responses.**

A predicted response, the output of radial basis function in (5), has a range of [0, 1]. To fit the predicted response vector $\boldsymbol{x}$ from (6) to the neural response vector $\boldsymbol{y}$, we used the linear transformation

$\tilde{\boldsymbol{x}}\boldsymbol{=}\left( \frac{\left| \boldsymbol{y}_{\mathbf{F}} \right|\boldsymbol{-}\left| \boldsymbol{y}_{\mathbf{NF}} \right|}{\left| \boldsymbol{x}_{\mathbf{F}} \right|\boldsymbol{-}\left| \boldsymbol{x}_{\mathbf{NF}} \right|} \right)\left( \boldsymbol{x}\boldsymbol{-}\left| \boldsymbol{x}_{\mathbf{NF}} \right| \right)\boldsymbol{+}\left| \boldsymbol{y}_{\mathbf{NF}} \right|$, (8)

where $F$ and $\mathrm{NF}$ denote the face and non-face stimuli. With this transformation, $\tilde{\boldsymbol{x}}$ and $\boldsymbol{y}$ shared the same average responses to both face and non-face stimuli. It should be noted that this linear transformation was conducted after searching for the optimal combination of $j$, $\alpha$, and $\boldsymbol{b}$, and it did not change either the global or local correlations.

**The identified visual features.**

We identified the visual features from 39 sites that exhibited significant response variations across the facial views (Fig. 5). We observed the same feature identified from multiple sites (e.g., the feature from S2_e2, S3_a3, S2_e1, S3_a2, and S2_d1 in Fig. 5). There are two possible reasons for representation of the same features in multiple sites. One reason can be that nearby sites encode the same visual feature. This could be the cases for S2_e1 and S2_e2, for S2_b3 and S2_b4, and for S3_a2 and S3_a3. These sites were located next to each other. It is not so surprising, considering the columnar structure and hierarchical organization in IT cortex^1,2^). More interestingly, we found that the identical features among 560K candidates were selected in the sites in two different monkeys. For example, S2_e1, S2_e2, and S2_d1 were derived from the monkey different from the monkey where S3_a2 and S3_a3 were derived. Similarly, S2_b3 and S2_b4 were from the different monkey where S3_a8 and S3_a4 were derived. Actually, S2 and S3 in site IDs stand for the monkeys where recordings were made from. In our previous paper, we also found that the same features derived from different monkeys^5^. These results suggest that there is a canonical feature set in high-level vision.

For supplementary information for readers, the figure visualizing the top five feature can be downloaded from the link: <https://github.com/YunjunNam0225/FragmentAnalysis_2021-02-11/blob/main/supplementary_figures/TopFiveFeatures_GITHUB.pdf>, and the figure for the features from 49 sites which were “not” significantly tuned by face view can be downloaded from the link: <https://github.com/YunjunNam0225/FragmentAnalysis_2021-02-11/blob/main/supplementary_figures/FeaturesFromNonViewTunedSites_GITHUB.pdf> .

**Contribution of color components.**

To investigate the contribution of color components for the prediction of IT neural responses, we conducted a test which only considers the orientation components for the prediction. That is, we defined another response set which utilizes the next equation to calculate the Euclidean distance instead of (3):

$\text{d}^{\text{2}}\boldsymbol{=}\sum_{\boldsymbol{o}} \left\| \boldsymbol{f}_{\boldsymbol{o}}\boldsymbol{-}\boldsymbol{s}_{\boldsymbol{o}} \right\|^{\boldsymbol{2}}$***.*  (9)**

Then, we conducted the same cross-validation test described in Fig. 4 in the main text except for that we evaluated the prediction performance of the responses to 532 face stimuli of the view tuned face columns (n = 39). With the features only considering the orientation, the prediction performance for 532 face stimuli (local correlation coefficient) was reduced to 0.116 ± 0.126 (Fig. S5). This value is lower than 0.369 ± 0.110 for the test with features consisting of both of local orientation and color components. We plotted scattergram where the vertical and the horizontal axes represent the Pearson’s correlation coefficient for the original (considering both orientation and color components) and new (considering only orientation components) predicted response vectors with the neural response vectors. This plot indicates that without the support of color components, the prediction performance drastically decreases from 0.37 to 0.17, proving significance of color components.

**Prediction of the view tuning curves.**

Following a search for the visual feature that explained the neural response to view uncontrolled faces and non-face objects, we utilized the feature to predict the view tuning curves drawn from the responses to the view-controlled faces (Fig. 2). As the measure to evaluate the similarity between the neural and predicted view tuning curves, the predicted responses were transformed into *z*-scores by (2), then the Pearson’s correlation coefficient were calculated between the two *z*-score vectors. We will refer to this correlation coefficient as *the predictability* (see Fig. 2 for the values from each site). Among 39 sites, 33 sites showed a predictability larger than 0.0973, which meant a significant correlation (α = 0.05) was found for the two view tuning curves.

**Prediction of the identity tuning curves.**

Next, we used the feature to predict the identity tuning curves drawn from the same response set. To draw the identity tuning curves, the predicted responses were transformed into z-score with the following equation, which discard response variation across views,

$\text{z}_{\text{iv}}\boldsymbol{=}\frac{\text{y}_{\text{iv}}\boldsymbol{-}\text{μ}_{\text{v}}}{\text{σ}_{\text{v}}}$, (10)

where $\mu_{v}$ and $\sigma_{v}$ are the mean and standard deviation of the 41 responses $([y_{iL}, \ldots, y_{iR}])$ from a single facial view $v$. After calculating the z-scores, the mean of $z_{iv}$ for each identity was averaged across seven views, then plotted as the identity tuning curves (the right side of Fig. 6A, 6B). In the plot, the averaged z-scores are drawn by the line, and their standard deviations are visualized by the shade. Note that the view tuning curves are drawn by averaging the z-scores of 41 identities in the same view, but the identity tuning curves are drawn by averaging the scores of seven views for each identity. We compared the identity tuning curves from actual and predicted responses (Fig. S6), and the significant Spearman’s correlations (p < 0.05) were observed from 13 sites (92.9%) among 14 sites showed significant response variation across face identities (p < 10^-6^, ANOVA).

**Visualization of the common facial features captured from a single facial view.** As the first step to explain the view tuning property, we found the sub-regions of face stimuli captured by each of the identified visual features. Then, we averaged the sub-regions for 41 individual faces from one facial view to obtain common facial features captured by the visual feature. The common facial features were visualized in the following way. (Fig. S7A). First, we collected face images from one facial view (step 1 in Fig. S7A), then resized and shifted them to align their facial structures (step 2). Face images were aligned vertically with respect to the vertical distance between the eyes and lips (the green line segment in Fig. S7A). Then, for the horizontal alignment, we fixed the positions of the left eye and tip of the left ear (both ends of the red line segments in Fig. S7A) for the left profiles (facial view = −90° and −60°), the positions of both eyes for the front-faces (−30°, 0°, and 30°), and the positions of the right eye and tip of the right ear for the right profiles (60° and 90°). After this alignment, the sub-regions were cropped from each stimulus (step 3), then their transparency level was adjusted to the predicted responses by alpha compositing ^10^ (step 4). In this step, the sub-regions from the stimuli with higher responses kept their visibility, but the sub-regions with lower responses became faded into gray. The adjusted images were overlapped to summarize the sub-regions captured by the visual feature from the facial view (step 5).

**Canonical correlation analysis (CCA) to visualize changes in the captured facial features essential to explain the view tuning.**

To address changes in the captured facial features with the horizontal rotation of faces, we investigated how their local orientation and color components changed when the faces were rotated from a non-preferred to a preferred view (Fig. S8). First, the sub-regions captured from 287 view controlled faces were represented by points ($\boldsymbol{S}\in\mathbb{R}^{D\times287}$) in a $D$–dimensional local orientation and color spaces ($D=H\times W\times7$). Then, we generated $\boldsymbol{l}_{\boldsymbol{V}}\boldsymbol{\in}\mathbb{R}^{287}$, whose elements had one-to-one correspondence with 287 face stimuli. Among these elements, we selected 41 elements corresponding to one of the seven views, then filled them with the mean predicted responses for the 41 individual faces matched with the elements. We repeated this procedure for another six views to fill $\boldsymbol{l}_{\boldsymbol{V}}$ with the mean responses for each view. Finally, one dimensional mapping ($\boldsymbol{a}_{\boldsymbol{V}}^{\top}\boldsymbol{S}$) for the sub-regions ($\boldsymbol{S}$) was obtained, which maximized the correlation with the generated vector ($\boldsymbol{l}_{\boldsymbol{V}}$), as follows,

$\boldsymbol{a}_{\boldsymbol{V}}\boldsymbol{=}\underset{\boldsymbol{a}}{\text{arg max}} \left\{ \text{corr}\left( \boldsymbol{a}^{\boldsymbol{\top}}\boldsymbol{S,}\boldsymbol{l}_{\boldsymbol{V}} \right) \right\}$. (11)

Maximizing the correlation allows $\boldsymbol{a}_{\boldsymbol{V}}\boldsymbol{\in}\mathbb{R}^{D}$ to be aligned with the direction from the points of the non-preferred view to those of the preferred view. Therefore, we could examine each element in $\boldsymbol{a}_{\boldsymbol{V}}$ to investigate how each component changed during the rotation. The $\boldsymbol{a}_{\boldsymbol{V}}$ also provided one dimensional representation ($\boldsymbol{a}_{\boldsymbol{V}}^{\top}\boldsymbol{S}$) for the sub-regions, where the information for the view tuning is maximally preserved (Fig. S8).

Please note that this equation was a special case of CCA, which received the pair of one matrix $(\boldsymbol{S)}$ and one vector $(\boldsymbol{l}_{\boldsymbol{V}}\boldsymbol{)}$ as an input, not like a general CCA receiving a pair of two matrices as an input. In our case, the linear transformation for the vector $\boldsymbol{l}_{\boldsymbol{V}}$ was found as a single scalar value which has no physical meaning. Therefore, this study will only deal with $\boldsymbol{a}_{\boldsymbol{V}}$, the linear transformation found for the matrix $\boldsymbol{S}$.

**Mechanisms for face columns used to acquire three different view tuning properties.**

We applied the explained methods to three sites with distinctive view tuning curves, which were tuned to front-faces (Fig. 7), tuned to right profiles (Fig. S10), and mirror symmetrically tuned to both the left and right profiles (Fig. S9). Here, we summarized how the visual features explained the view tuning properties observed from the eight example sites (see Fig. S11).

As we already discussed in Fig 7, site S1_a has the response tuned to front-faces. The visual feature for this site originated from the image fragment of a red snack box, and it attempted to capture the facial region including the eyes and lips. The horizontal components of these facial parts in the front view were well matched with the feature. However, with the horizontal rotation from front to left and right views, these facial parts gradually disappeared behind the sight, and the area occupied by the horizontal components began to shrink. Therefore, the matching capacity with the visual feature reduced, explained the decrease in the predicted responses toward profile views.

In site S1_b, the visual feature originated from an image fragment of a man wearing a pink hairband (Fig. S11). The predicted responses from this feature were maximum for the front-face, because they have two hairlines, which enclose the facial area from the left and right sides. Depending on identities, the sub-regions with the best match were found from the forehead area above the eyes, or the cheek and nose area below the eyes. During the rotation toward profiles, one of these two hairlines disappeared behind sight, which explained the lower responses found in the profiles.

In site S1_f, another site tuned to front-face, the visual feature originated from images of a luxurious ceiling light, whose cage evoked multiple orientation components (Fig. S11). Due to the orientation components, the feature captures a global structure of front-face. However, the matching capacity with the feature decreased toward profile views, because with the rotation of faces, the areas occupied by the eyes and mouth become narrower making the faces lose orientation components similarly to the case of site S1_a.

S2_e2 showed that the responses were tuned to right profiles (Fig. S11; see also Fig. S10). The visual feature identified from this site originated from the fragment of a forehead image. The sub-regions appropriately matched with the feature were found along the hairline in the right profiles. However, in the left profiles, where black hairs were located on the right side of the facial region, optimal matches for the sub-regions could not be found. That is, the selectivity for right profiles observed in this site could be explained by the existence of the hairline with the specific color arrangement, in which the area of black hairs was located on the left side of the area of facial skin.

Site S2_h6 also showed preferential responses to right profiles (Fig. S11). The feature was generated from the image fragment of a chair that could be characterized by 1) the left area with the black color, 2) the right area with yellowish color and complex structures (local orientations in 0° and 90°), and 3) the borderline in 90° dividing the two areas. This feature was well matched with the right profiles, because they have the area of black hairs on the left side and the area of yellowish skin with complex structures (of the right eye) on the right side. In the left profiles, the eyes and hairs are arranged in opposite directions, therefore they have a weaker matching capacity with this feature.

The feature obtained from site S3_a5 also could be characterized by a borderline separating the two areas with different colors (Fig. S11). The borderline induced a strong orientation component at 45°, and the right area had local orientations at 0°. The feature was well matched with the entire area of right profiles, but in the left profiles, the sub-regions with good match could not be found.

The last two sites (S2_r5 and S2_r7) showed the mirror symmetric selectivity on both profiles (Fig. S11; see also Fig. S9). The features from two sites captured the facial region and vertically elongated it from the chin over the hairline and horizontally located it between the eye and ear. The pattern within this region (the upper black and lower skin color areas without orientation components) could be commonly found from both profiles but could not be found from the front-faces. Although the frontal faces had the color arrangement of the black area above the skin color area, the lower area was filled with local orientation components from the facial parts (Fig. S9). This difference explained the lower responses to front-faces.

**Linear combination of the view-tuned features to establish view invariant face representation.**

In relation to the view invariant representation of faces by population activity, we tested whether faces from different identities could be separated in high dimensional space where each axis was defined by predicted responses from the 29 visual features identified from the 39 view-tuned sites. We have 39 recording sites tuned to particular facial views, but we found the same feature identified from multiple sites. Here, the same feature refers to two or more visual features that originated from the same natural image fragment (sharing the same value of $j$). To avoid the analysis being biased to duplicated features, we selected the feature with the highest $b_{\max}$ and $\alpha$ values among the duplicated ones and discarded the others.

Then, we divided the 287 faces (7 views × 41 identities) into seven faces from one target identity and 280 faces from 40 non-target identities. Let us define the predicted responses for the target faces as $\boldsymbol{X}_{T}\boldsymbol{\in} \mathbb{R}^{29\times7}$, and the responses to the non-target faces as $\boldsymbol{X}_{N}\boldsymbol{\in} \mathbb{R}^{29\times280}$. By concatenating two matrices, we obtained $\boldsymbol{X=}\left[ \boldsymbol{X}_{T}\boldsymbol{,}\boldsymbol{X}_{N} \right]$. Let us define $\boldsymbol{l}_{\mathrm{ID}}\boldsymbol{\in} \mathbb{R}^{1\times287}$, the binary labels for $\boldsymbol{X}$, whose first seven elements (corresponding to the target identity) are 1, but later elements are 0. To search for the axis maximally separating $\boldsymbol{X}_{T}$ and $\boldsymbol{X}_{N}$, we searched for the projection vector $\boldsymbol{w}$ such that

$\boldsymbol{w}\mathbf{=}\underset{\tilde{\boldsymbol{w}}}{\text{arg min}} \left( \left\| \boldsymbol{l}_{\mathbf{ID}}\mathbf{-}{\tilde{\boldsymbol{w}}}^{\boldsymbol{\top}}\boldsymbol{X} \right\| \right)$. (12)

For each of the 41 identities, we calculated $\boldsymbol{w}$, then investigated representations for target $(\boldsymbol{w}^{\top}\boldsymbol{X}_{T}\mathbf{)}$ and non-target identities $(\boldsymbol{w}^{\top}\boldsymbol{X}_{N}\mathbf{)}$ how much they were separable to each other (Fig. S12A). To evaluate the separability, we drew ROC curves regarding $\boldsymbol{w}^{\top}\boldsymbol{X}_{T}$ and $\boldsymbol{w}^{\top}\boldsymbol{X}_{N}$ as positive and negative classes of a binary classification problem (Fig. S12B). In five of the 41 identities, target faces were perfectly separated from the non-target faces (AUC = 1.0, the red line in Fig. S12B), and the average AUC (area under the ROC curve) value for the 41 identities was 0.992 ± 0.014 (minimum value = 0.923; see Figs. 8A and S12B). We conducted the same analysis for the neural space, where each axis was defined by the actual columnar responses recorded from the 39 view-tuned sites (Fig. S12C). In three identities, target faces were perfectly separated from the non-target faces, and the mean of AUC was 0.981 ± 0.023 (minimum value = 0.922; see Figs. 8A and S12D).

We tested the generalization performance using the leave-one-view-out test. For a single test, we selected one of 287 faces, taken from one facial view of the $i$-th target identity. Then, 41 faces sharing the same view were set aside as the test set, and the remaining 246 faces were assigned to the training set. From the training set consisting of six faces from the $i$-th target identity and 240 faces from other identities, we obtained $\boldsymbol{w}$ by (7) to separate these two groups of faces in the feature space. Then, the predicted responses from 41 faces in the test set were projected onto $\boldsymbol{w}$, and we tested whether the projected value for the face of the $i$-th identity was larger than 40 values for other identities. This test verified that $\boldsymbol{w}$ can correctly pick out the target identity among the faces taken from a new facial view. We repeated this test by replacing the facial view and target identity, and the ratio of successful identification rate was measured as 65.9% (= 189 among 287 faces, chance level = 1/41 ≈ 2.4%).

**Searching visual features for the deep convolutional neural network (DCNN)**

We also investigated whether the fragment-based approach could identify visual features encoded in the “artificial” deep neural networks. Among the various architectures, we selected a convolutional neural network VGG-16^11^ for its simple architecture and the reasonably high score in predicting a neural response^12^.

In VGG-16, each layer consists of the nodes with different numbers of width, height, and depth. In this network architecture, all nodes in the same depth slice (an activation map) share the same convolutional kernel which encode different receptive fields. Therefore, we attempted to identify the visual features encoded by each kernel, instead of each node. To obtain the responses for each kernel, we fed 1,509 face and non-face stimuli (Fig. S1) into the network and collected the activations of all nodes. Then, we pooled the maximum of all nodes in the same depth slice. For example, in the case of the thirteenth convolutional layer having 100,352 nodes (width: 14, height: 14, depth: 512), we collected the activations of 196 (= 14 × 14) nodes involved with a single kernel, then pooled their maximum value to obtain a response for the kernel. For each kernel, we collected the responses for 1,509 stimuli as one response vector, then searched for the visual feature that best described this vector, as we did for the neural responses.

Among the 16 layers (13 convolutional and three fully connected layers; note that five max pooling layers were not included) of VGG16, we attempted to identify the visual features encoded in the thirteenth layer, the last convolutional layer before the fully-connected ones, due to its highest face-selectivity index (FSI). Since the fragment based approach was developed for analyzing responses from the face columns, it would be reasonable to apply this method to the layer with the same response property, namely the high FSI (see Fig. S14 for the FSI measured from each response vector collected from kernels from thirteen convolutional layers). We searched for the visual feature that best described the kernel response vectors for 512 kernels as we did for the neural responses. The average of the correlation coefficients between the predicted and actual response vectors were 0.420 ± 0.130 (max: 0.717, p < 0.05 in 510 among 512 sites). This result suggested that the fragment-based approach could provide visual features encoded in the higher layers of DCNN.

References

1 Sato, T., Uchida, G. & Tanifuji, M. Cortical columnar organization is reconsidered in inferior temporal cortex. *Cerebral cortex.* **19**, 1870-1888 (2009).

2 Sato, T. *et al.* Object Representation in Inferior Temporal Cortex Is Organized Hierarchically in a Mosaic-Like Structure. *J. Neurosci.* **33**, 16642-16656 (2013).

3 Tsao, D. Y., Freiwald, W. A., Tootell, R. B. & Livingstone, M. S. A cortical region consisting entirely of face-selective cells. *Science.* **311**, 670-674 (2006).

4 James, M. N. & Jane, M. C. *Statistics and Chemometrics for Analytical Chemistry*. (Pearson education, 2010).

5 Owaki, T. *et al.* Searching for visual features that explain response variance of face neurons in inferior temporal cortex. *Plos ONE.* **13**, 1-27 (2018).

6 Everingham, M., Luc, V. G., Williams, C. K., Winn, J. & Zisserman, A. The PASCAL Visual Object Classes (VOC) Challenge. *Int. J. Comp. Vis.* **88**, 303-338 (2010).

7 Serre, T., Wolf, L., Bileschi, S., Riesenhuber, M. & Poggio, T. Robust Object Recognition with Cortex-Like Mechanisms. *IEEE T. Pattern. Anal.* **29**, 411-426 (2007).

8 Mutch, J., Knoblich, U. & Poggio, T. CNS: a GPU-based framework for simulating cortically-organized networks. *MIT-CSAIL-TR-2010-013, CBCL-286* (2010).

9 Ito, M., Tamura, H., Fujita, I. & Tanaka, K. Size and position invariance of neuronal responses in monkey inferoterotemporal cortex. *J of Neurophys.* **73**, 218-226 (1995).

10 Porter, T. & Duff, T. Compositing Digital Images. *SIGGRAPH Computer Graphics* **18**, 253-259 (1984).

11 Simonyan, K. & Zisserman, A. Very Deep Convolutional Networks for Large-Scale Image Recognition in *International Conference on Learning Representations (ICLR).*

12 Schrimpf, M. *et al.* Brain-Score: Which Artificial Neural Network for Object Recognition is most Brain-Like? *bioRxiv preprint* (2018).

13 Lin, T.-Y. *et al.* in *13th European Conference on Computer Vision.* (eds D Fleet, T Pajidla, B Schiele, & T Tuytelaars) 740-755 (Springer, Cham).

**Figures**


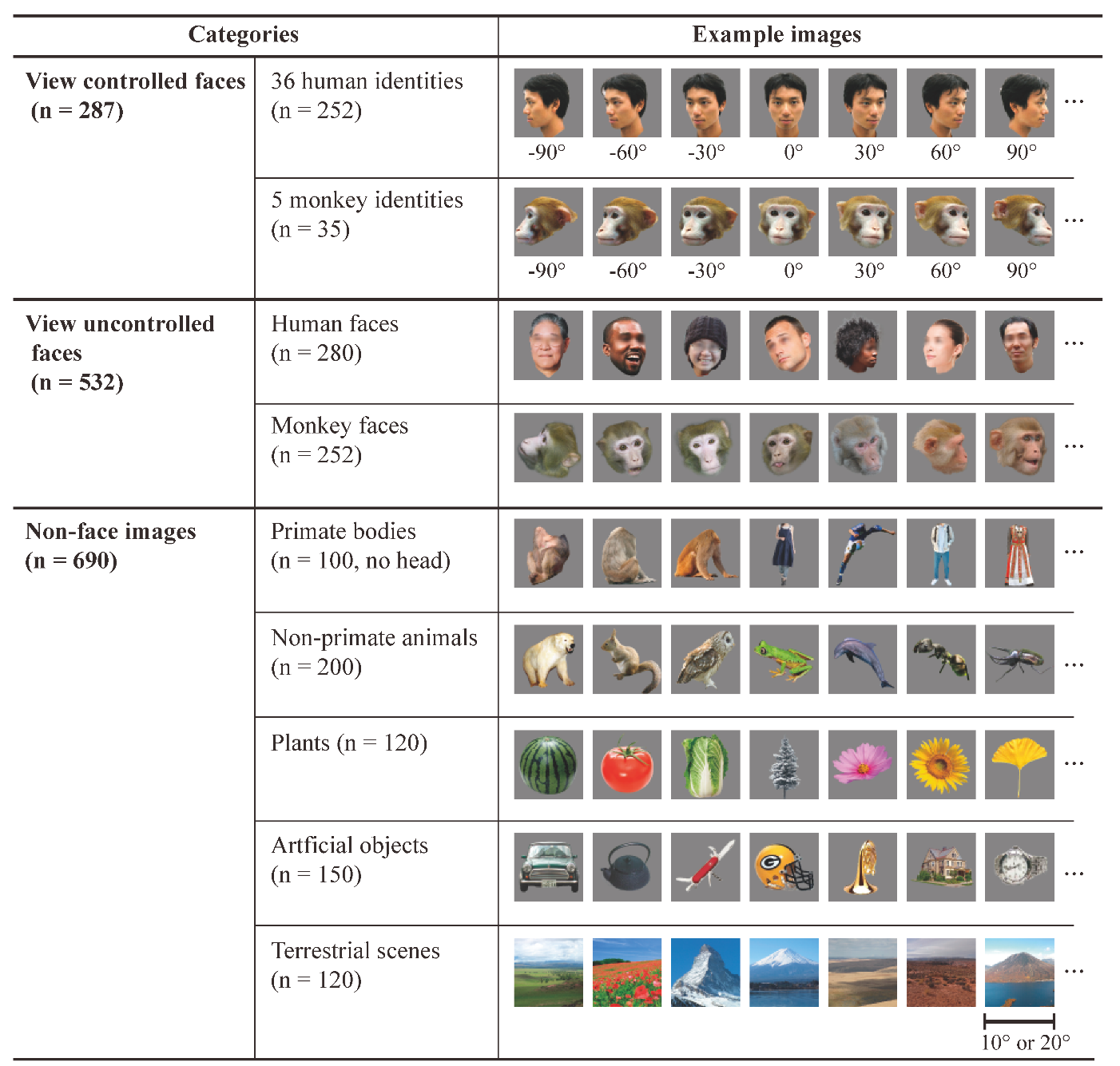


**Fig. S1.** 1,509 visual stimuli consisting of 287 view controlled faces, 532 view uncontrolled faces, and 690 non-face objects. The view controlled faces comprised the faces taken from seven facial views—every 30˚ from the left (−90˚) to the right profile (+90˚)—of 41 identities (36 humans and five monkeys). However, the view uncontrolled faces were cropped from randomly selected human (n = 280) and monkey (n = 252) faces, creating a large variety of head orientations. There was no overlap of identities between the view uncontrolled faces and the view controlled faces. The eye regions of the view uncontrolled human faces were blurred for copyright reasons.


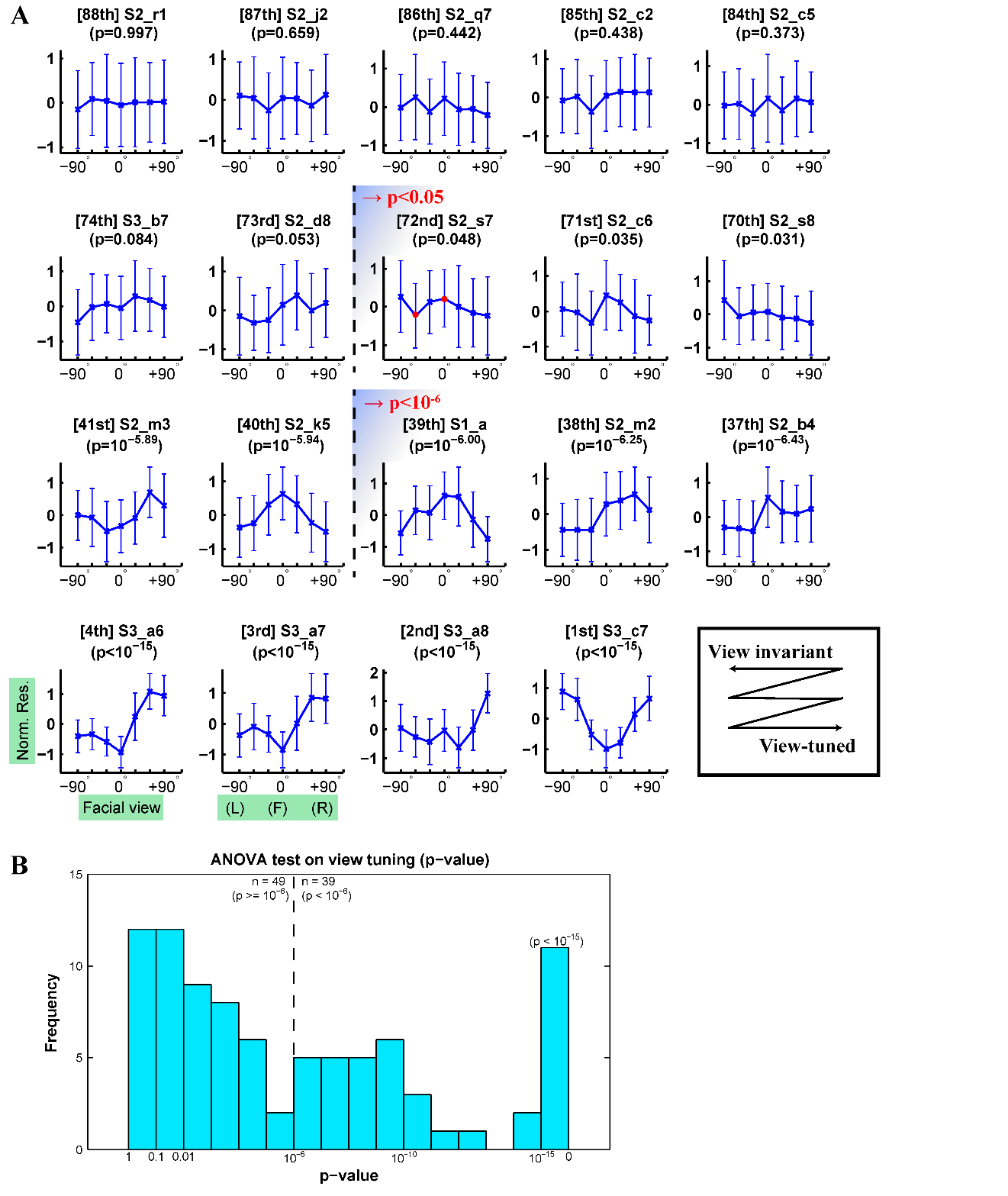


**Fig. S2.** ANOVA test to select the sites whose responses were tuned to particular facial views. (A) The view tuning curves from the example sites were sorted by p-values from the ANOVA test, in which the NULL hypothesis was “the mean responses for seven views that were the same.” The curves became more specific to particular views with the decrease of p-values. We used 10^-6^ as the threshold to select out view-tuned sites (third row), instead of the conventional cutoff level 0.05 (second row). (B) histogram for the p-values from the reliable and face-selective sites (n = 88). With the threshold of 10^−6^, 39 sites were categorized as view-tuned sites.


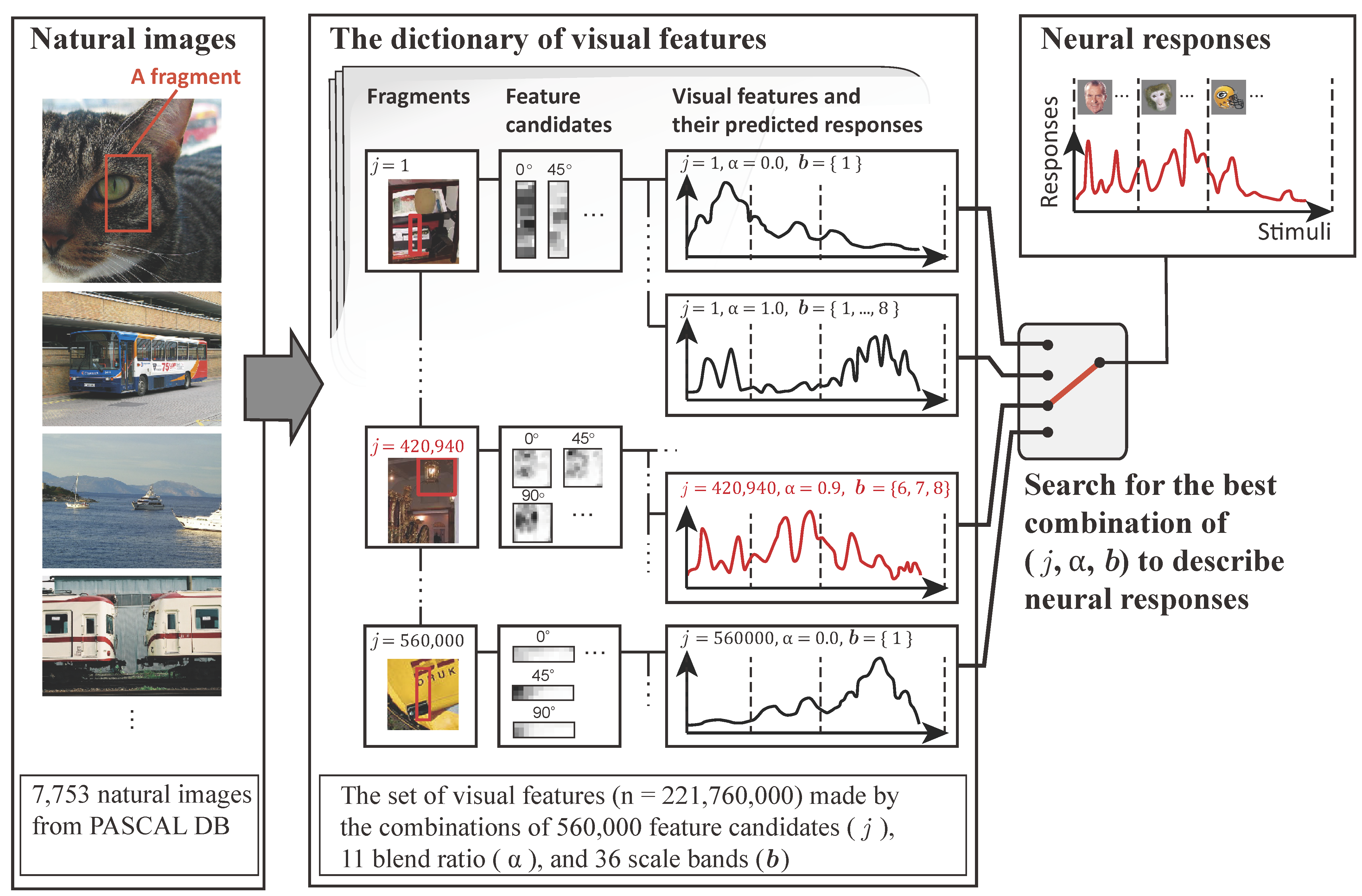


**Fig. S3.** Searching for the visual features from the dictionary of image fragments. Natural images from the PASCAL image database were cut into 560,000 image fragments. From each fragment, 396 visual features (11 blend ratio × 36 scale bands) were generated. Among the massive number of visual features (n = 221,760,000), we searched for the best feature, whose predicted responses showed the maximum correlation with the neural responses recorded from a single column.


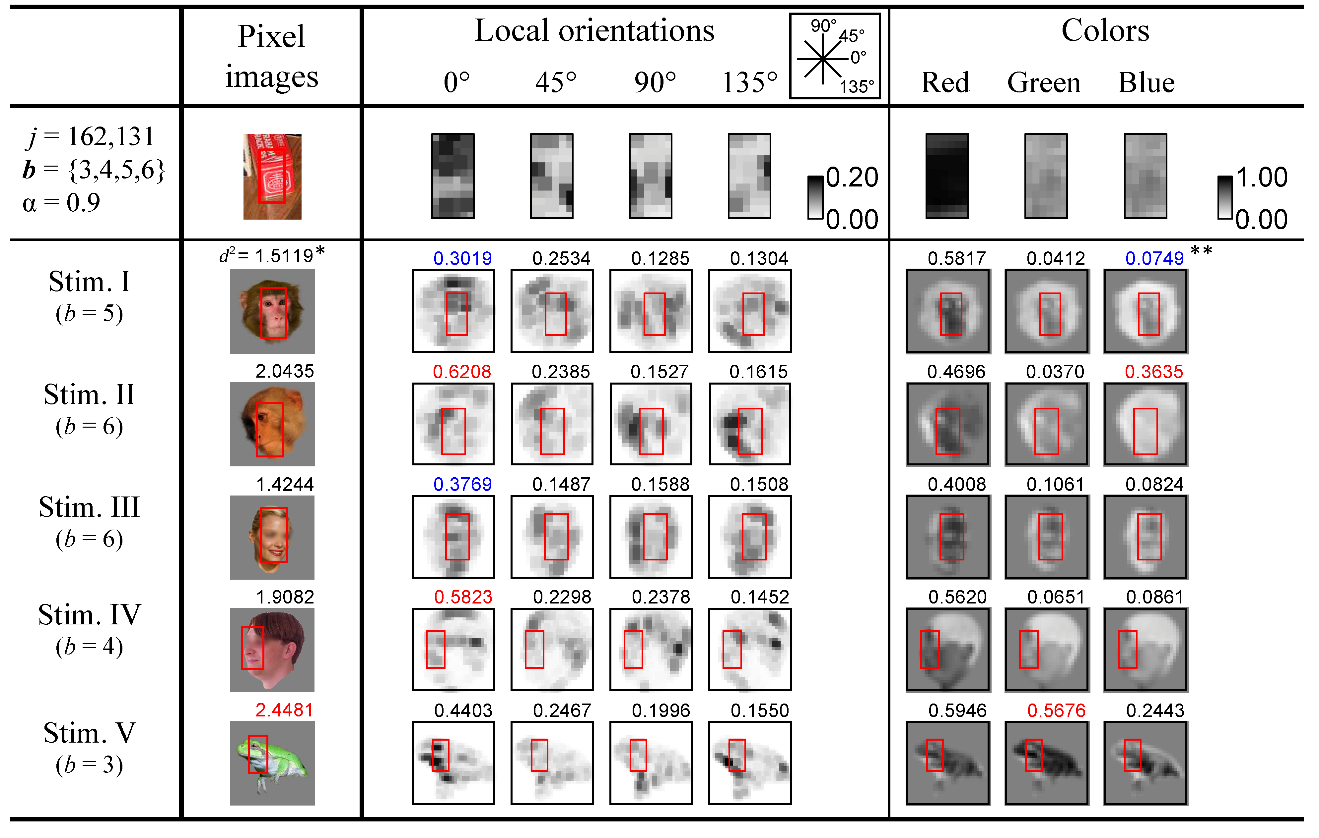


| * | $\boldsymbol{d}^{\boldsymbol{2}}$ is the sum of seven squared distances measured from each channel. |
| --- | --- |
| ** | The squared distance between candidate and stimulus.  In orientation channels (third column), computed by $\alpha\left\Vert\boldsymbol{f}_{o}\boldsymbol{-}\boldsymbol{s}_{o} \right\Vert^{\boldsymbol{2}}$.  In color channels (fourth column), it is computed by $(1-\alpha)\left\Vert\boldsymbol{f}_{c}\boldsymbol{-}\boldsymbol{s}_{c} \right\Vert^{\boldsymbol{2}}$. |

**Fig. S4.** The example feature candidate (in the second row) was characterized by a combination of reddish color with horizontal orientation components, which coincided with the facial configuration made of eyes, nose, and mouth. Its response variations among the faces could be explained as follows. Compared to the first stimulus (the monkey face in the front view), the second one has a tilted face with brownish color, which causes the sub-region to have weaker components in the 0° (horizontal) and blue channels. Due to this difference, the second stimulus showed a larger distance in the 0$^{\circ}$ (= 0.6208) and blue (= 0.3635) channels than those (0.3019 and 0.0749) from the first stimulus. In the third and fourth stimuli, the candidate captured the facial area with strong horizontal components. However, the facial area in the fourth stimulus was too narrow to fit with the candidate, which caused the left part of the sub-region to be deviated from the facial area. This difference made the fourth stimulus distance in the 0° channel (= 0.5823) larger than the third stimulus (= 0.3769). In the frog image of the fifth stimulus, the candidate was able to indicate the facial region of the frog. However, due to the strong green components (the distance in the green channel was 0.5676), which was the largest distance ($d^{2}$ = 2.4481) measured among the five stimuli. The eye regions of stimulus III and IV are blurred for copyright reasons.


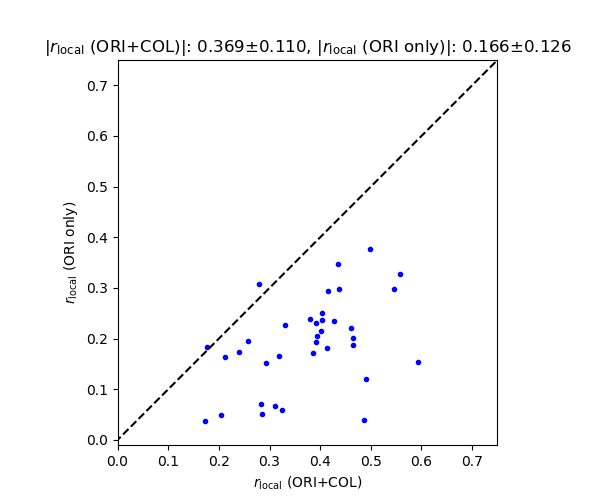

**Fig. S5.** The contribution of color components for the prediction of neural response variance of view tuned face columns (n = 39). The two-fold cross-validation test to predict the responses to face stimulus set ($r_{\text{local}}$).


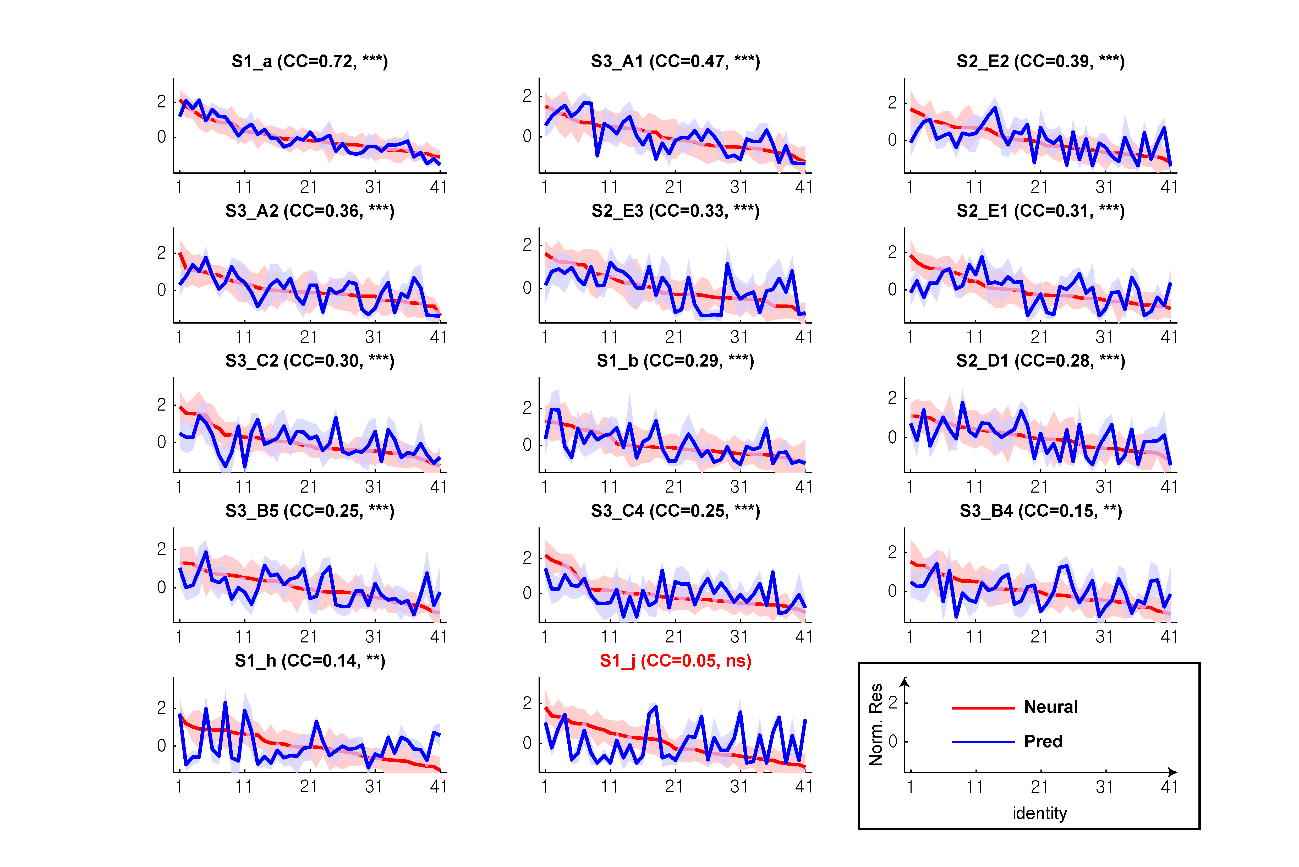


**Fig. S6.** Actual (red) and predicted (blue) identity tuning curves from 14 recording sites. Mean (line) and standard deviation (colored shade) across seven views are plotted after taking a z-score. The sites are sorted by the Spearman’s rank-correlation coefficient between two identity tuning curves. Among fourteen sites, significant correlation (p < 0.05) was found from thirteen sites (= 92.9%, the site IDs are in black). *: p < 0.05, **: p < 0.01, and ***: p < 0.001.


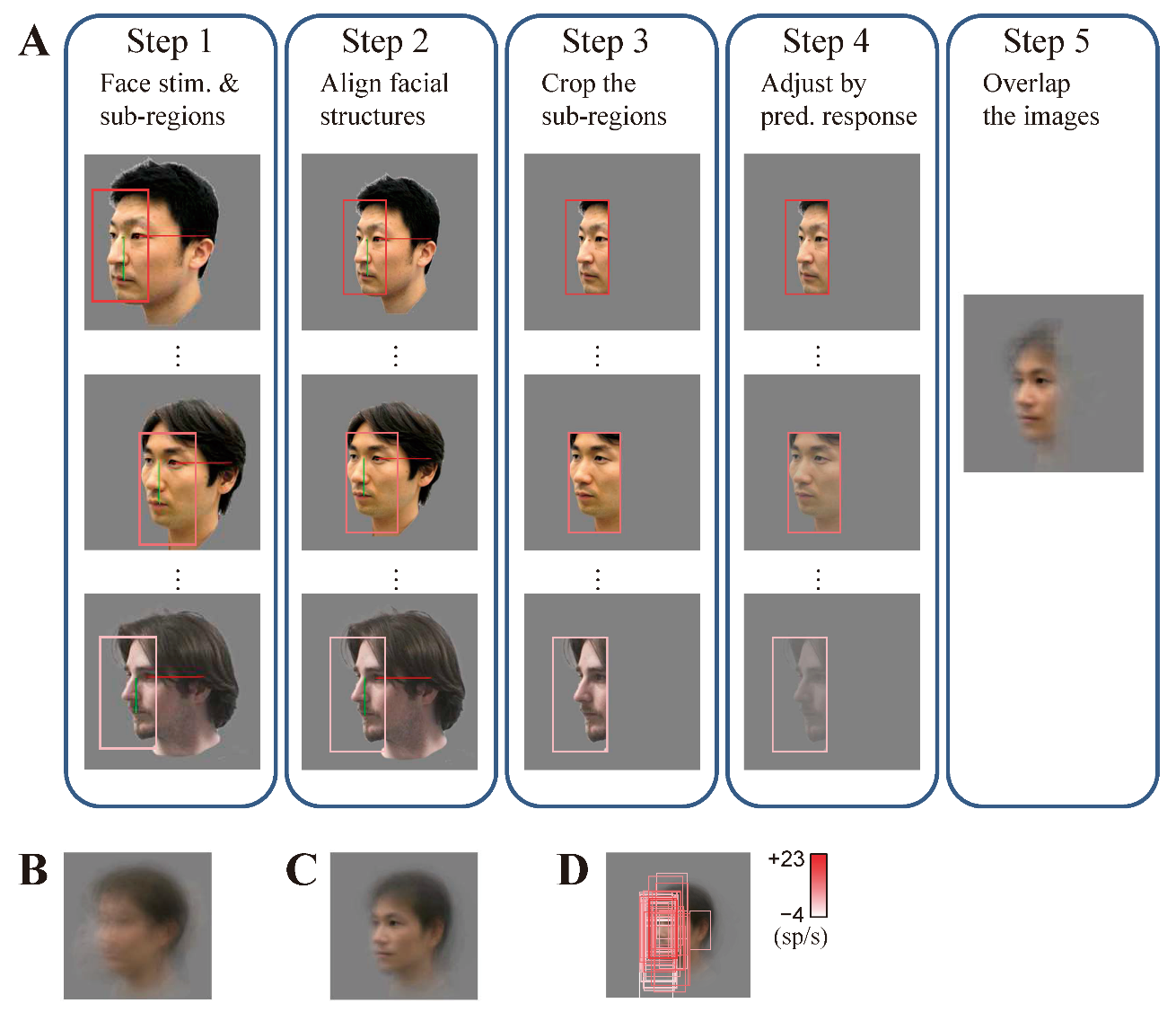


**Fig. S7.** (A) Five steps to average the sub-regions captured from an example facial view (−60°). See Fig. 7a for the visual feature. (B) The average of 41 face images in step 1 before the alignment procedure. (C) The average of 41 faces in step 2 after the alignment. (D) The sub-regions captured from each of the 41 faces are indicated by rectangles. The color of rectangles denotes the predicted responses generated from each of sub-regions. The five step procedure depicted in (A) visualizes the part of the faces commonly captured by the feature from the 41 faces with the same facial view.


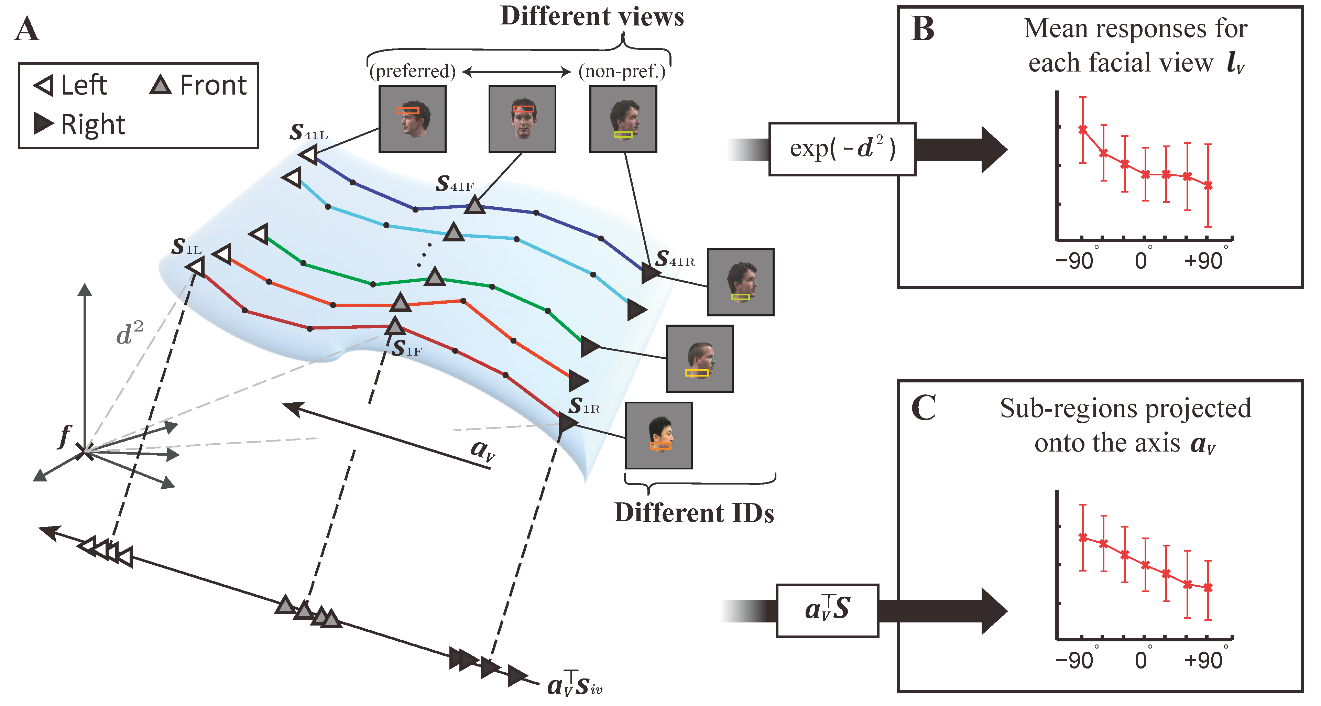


**Fig. S8.** We utilized CCA to identify the axis that best described the change in the capture sub-regions when the faces were rotated from a non-preferred to preferred view. (A) Let us consider a $D$–dimensional local orientation and color space, where the sub-regions from 287 faces (41 identities × 7 views) were represented as the points ($\boldsymbol{S}\in\mathbb{R}^{D\times287}$) with the same number (see (A)). Let us also assume that the responses from $\boldsymbol{S}$ were maximum to the left profiles, then gradually decreased toward the right profiles (see the tuning curve in (B)), the points for the left profiles ($\boldsymbol{s}_{1L},$ …, $\boldsymbol{s}_{41L}$) were more closely located to the visual feature (smaller $d^{2}$ from $\boldsymbol{f}$) than the points for the right profiles ($\boldsymbol{s}_{1R},$ …, $\boldsymbol{s}_{41R}$). Our interest was to identify the axis that best described the change within the sub-regions when the faces were rotated from the non-preferred (right) to the preferred (left) view. To solve this problem, we sought the axis $\boldsymbol{a}_{\boldsymbol{V}}$ such that the projection onto the axis ($\boldsymbol{a}_{\boldsymbol{V}}^{\top}\boldsymbol{S}_{\boldsymbol{iv}}$) achieved the maximum correlation with $\boldsymbol{l}_{\boldsymbol{V}}$ (the vector was filled with mean responses for each facial view). The CCA seeks the one dimensional representation for the sub-regions, where the two view tuning curves (the mean values for each facial view) from predicted responses (see (B)) and projection results (see (C)) were maximally correlated.


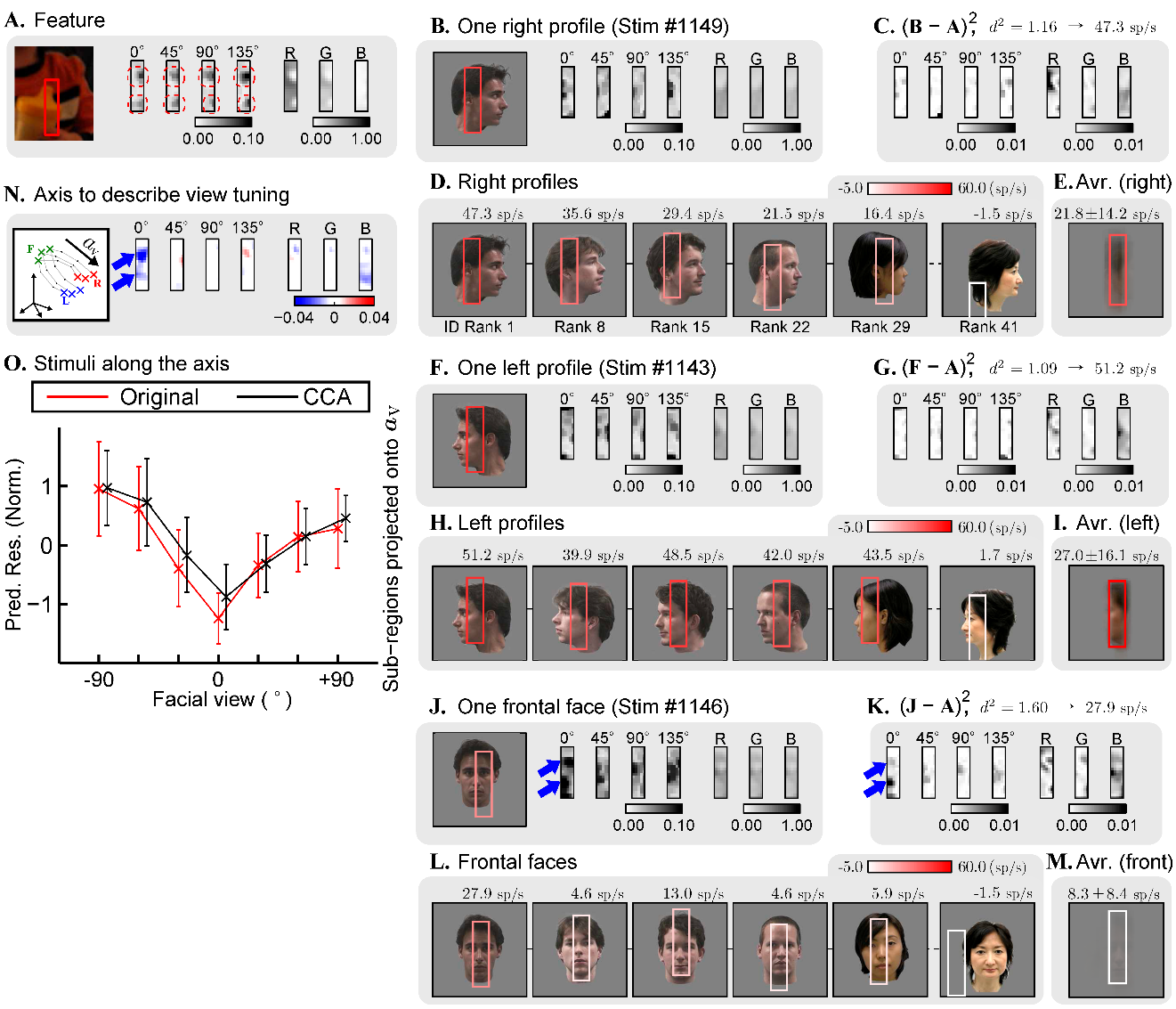


**Fig. S9.** (A) The identified visual feature of site S2_r5 with the mirror symmetric view tuning. The feature, originated from an image fragment of a feeding bottle, was characterized by local orientations demarcated by red broken lines. (B) The face with the largest predicted response among the right profiles, and the sub-region captured by the feature (red rectangle). (C) Within the sub-region, the orientation components from the hairline were well matched with the feature, thus the squared distance between (B) and (A) was small (= 1.16). (D)-(E) The sub-regions captured from the right profiles (D) and their weighted average (E). These sub-regions were characterized by a hairline dividing upper (black) and lower (skin color) areas. (F)-(I) The same as (B)-(E), but with the left profiles. The feature also captured the sub-regions and vertically elongated around the hairline. (J) The front-face of the identity shown in (B) and (F). Compared to (B)-(F), the sub-region had strong horizontal components (blue arrow) because of the facial parts, such as the eyes and lips. (K) Due to the horizontal components, the front-face has a squared distance (= 1.60) higher than those from the profile faces. (L) The sub-regions captured from the front faces showed weaker predicted responses than the profiles. (M) Due to the weak responses (= 8.3 ± 8.4 sp/s), the weighted average becomes faint. (N) The axis identified by CCA indicates that the weak horizontal components in the profile faces are the factor determining their higher predicted responses. (O) The sub-regions projected onto the axis showed 0.740 (p < 10^−6^) of correlation with the original view tuning curve.

**
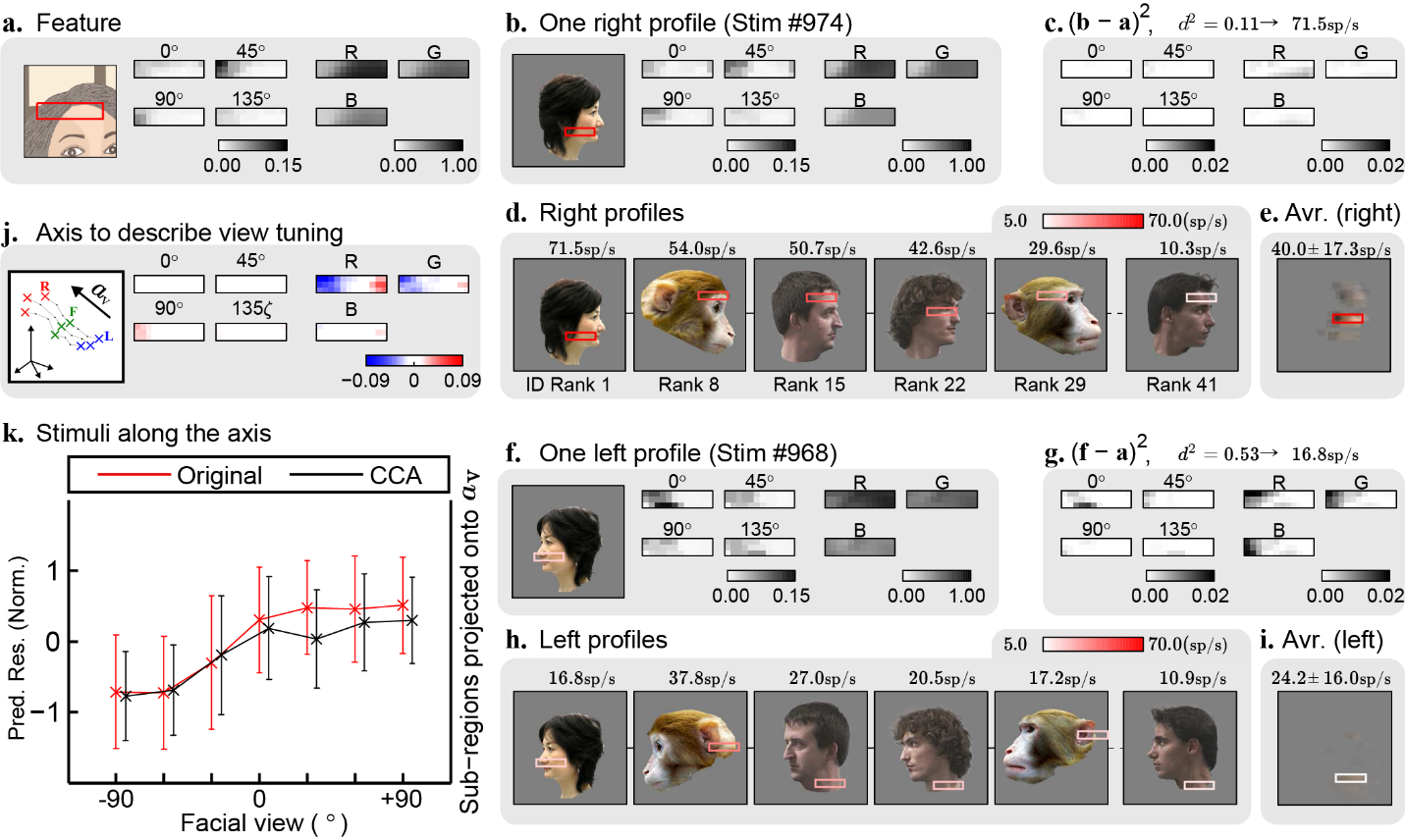
**

**Fig. S10.** (A) The identified visual feature of site S2_e2 whose responses tuned to right profiles. The fragment was found from the picture of the forehead where the left (black hair) and right (facial skin) areas are divided by the hairline inclined to the right side. (B) In the face with the highest predicted response, the sub-region was found on the hairline behind the mouth (red rectangle). (C) The difference between (B) and (A) was small across all components ($d^{2}$= 0.11). (D) The sub-regions from the other right profile faces were located along the hairline. (E) The weighted average of the sub-regions from the right profiles, which points out the areas along the hairline. (F) The sub-region captured from the left profile of the same identity shown in (B). In the hairline of the left profile, black hairs located at the right side of the facial region. Therefore, the hairline in this face cannot be matched with the feature, and the sub-region was located on the face line around the nose. (G) The difference between the feature and the sub-region shown in (F). The distance (= 0.53) was higher than the right profile (= 0.11) because of the difference at the left side of the color channels. (H) The sub-regions from the left profiles of the same identities shown in (D). (I) The weighted average of the sub-regions from 41 left profiles showed a fainter image than (E), due to their smaller predicted responses (24.2 ± 16.0 sp/s). (J) The axis from CCA indicates that while the rotation from the non-preferred (left) to preferred (right) view, the color at the left side of the sub-regions changed from yellow to black (decrease in red and green channels). It suggested that the hairline with the specific color arrangement (black at the left side, and skin color at the right side) is the reason for the higher responses in the right profiles. (K) The sub-regions projected onto the axis showed 0.524 (p < 10^−6^) correlation with the original view tuning curve.


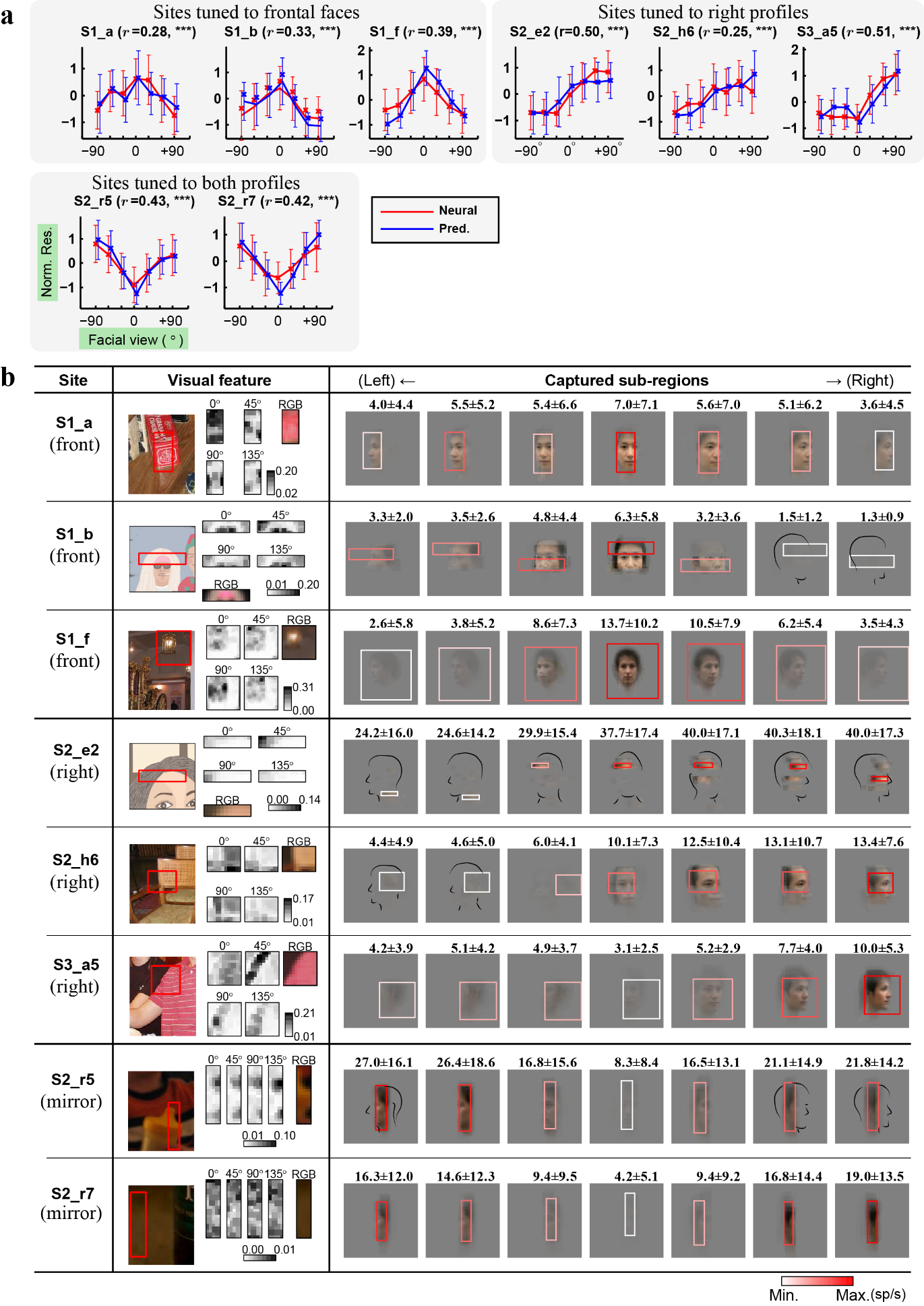


**Fig. S11.** Additional examples of how the visual features explained the view tuning properties. (A) View tuning curves from eight of the example sites. (B) Visual features identified from each site (second column) and weighted average of the sub-regions captured from each facial view (third column). The mean and standard deviation of the predicted responses are denoted over each image. The color of a rectangle indicated the mean predicted response for each view (was how large) among the seven facial views. The black lines are the hairline or face line of the averaged faces (for example, Fig. S7C) drawn to indicate the relative position of the sub-regions over the face stimuli.


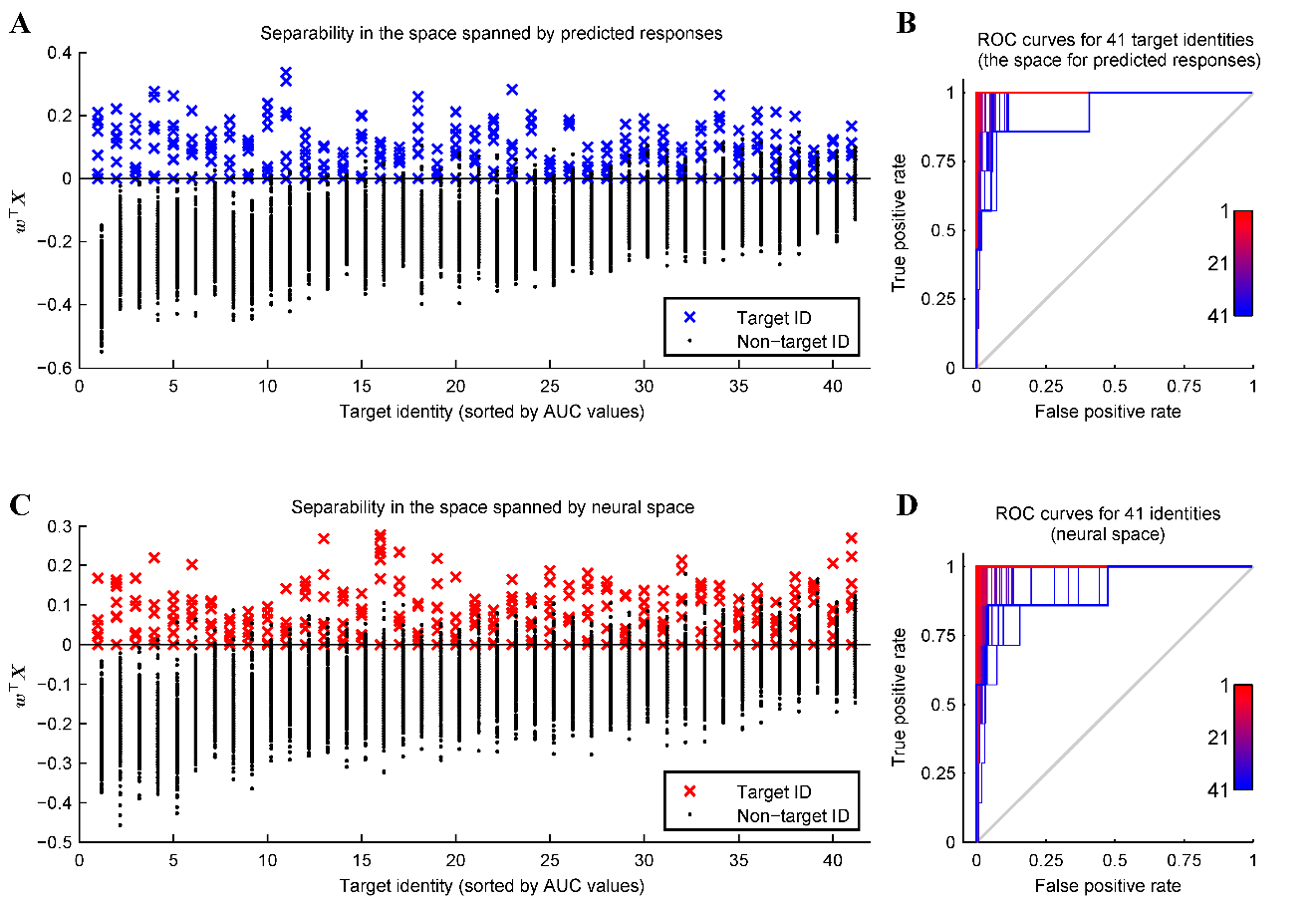


**Fig. S12.** (A) The seven faces of one target identity and 280 faces of non-target identities represented along the axis $\boldsymbol{w}$. Here, $\boldsymbol{w}$ is searched to maximize the separability in the space spanned by the predicted responses from 29 visual features. The horizontal axis denotes each pair of one target and 40 non-target identities. The minimum among the seven values for the target faces was set as the zero level. (B) ROC curves drawn from each pair of target and non-target identities. Each line with different colors indicates a single pair of target and non-target identities. (C)-(D) The same results obtained from the high dimensional space spanned by columnar responses from 39 recording sites.


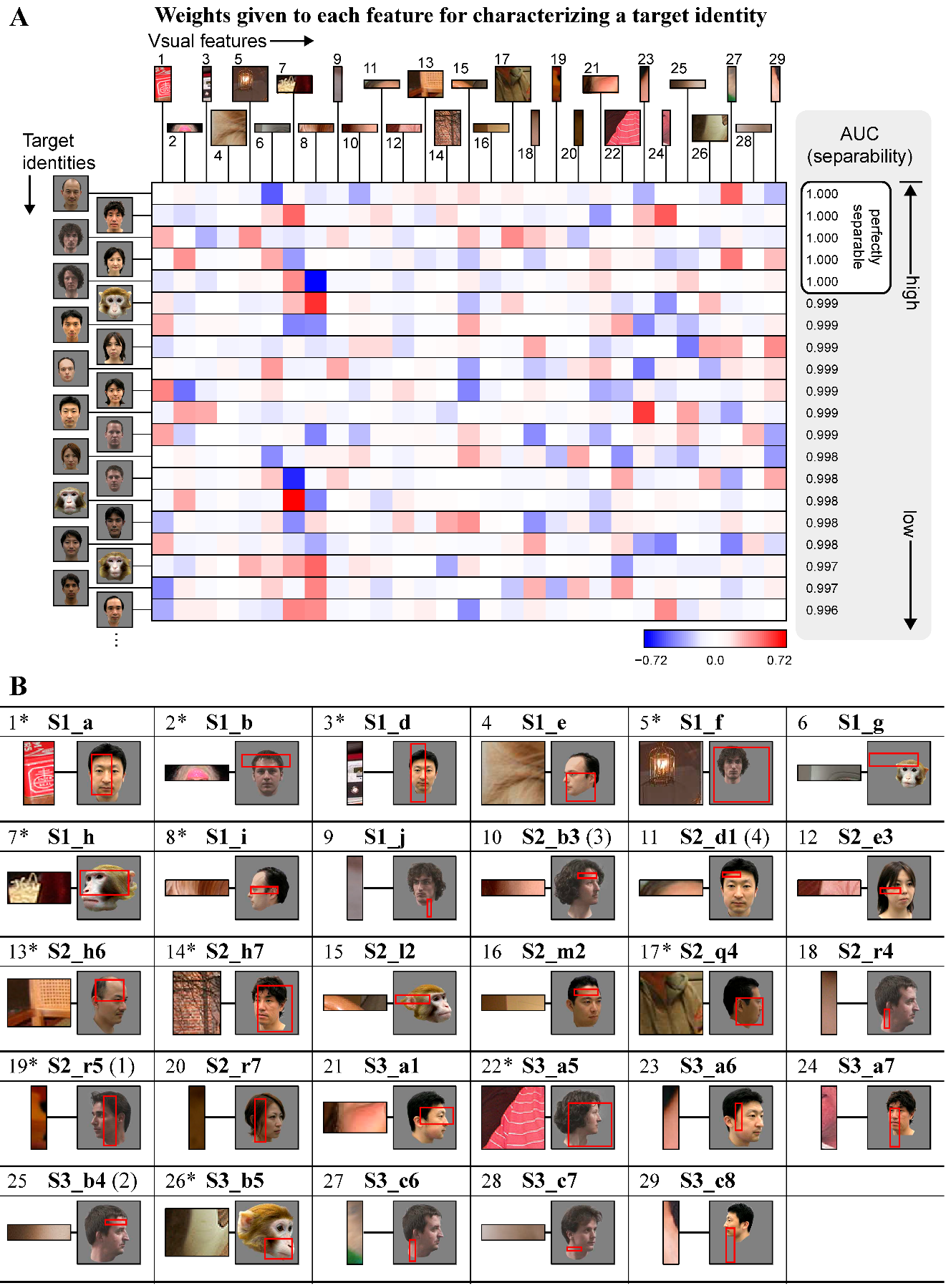


**Fig. S13.** (A) The weights in $\boldsymbol{w}$ given to each visual feature (the image fragments for each feature were depicted at the top) to separate the target identity (shown at the left side) from other non-target identities. The features with high absolute values indicate that they are key features to separate the target identity. (B) For each feature in (A)**,** we search for the face image with the highest predicted response with indicating the sub-region captured by the feature. The name of site where the feature was identified is also denoted. The number inside the bracket indicates the number of other sites where the same feature candidate was identified. *: 12 sites categorized as encoding configural features (see Discussion).


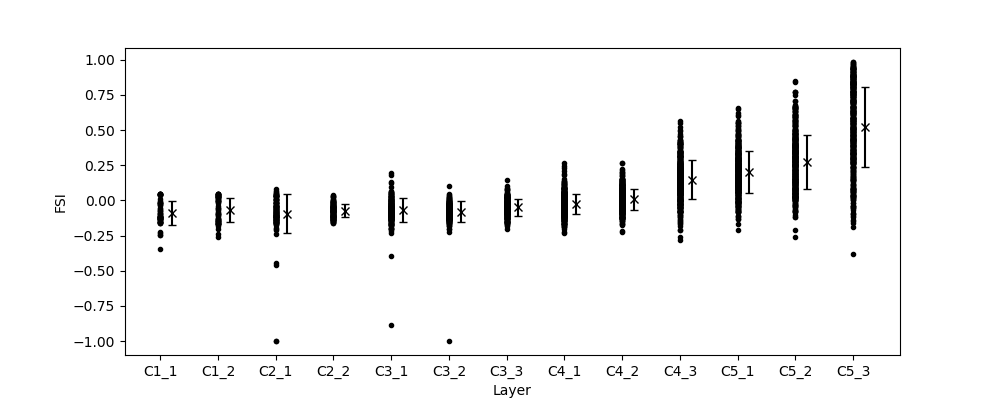


**Fig. S14.** The FSI of each kernel measured from thirteen layers of VGG-16 network. We collected the kernel responses (the maximum of activations from each depth slice) for 819 face and 690 non-face object images, then evaluated their FSIs. The FSIs measured from each kernel is denoted by the dots, and the mean and standard deviation for each layer are plotted by the error bars.

**Table S1.** Eight parameter settings (referred to the bands) to consider scale invariance of IT neurons. In higher bands, each component in a visual feature encodes larger area of a stimulus image. ^1)^ height × width, ^2)^ standard deviation of the Gaussian envelope, ^3)^ the wavelength of the sinusoidal factor, ^4)^ the same value was used for both of vertical and horizontal axes

| Band | | 1 | 2 | 3 | 4 | 5 | 6 | 7 | 8 |
| --- | --- | --- | --- | --- | --- | --- | --- | --- | --- |
| Gabor filter | Filter size^1)^ | 7 × 7 | 11 × 11 | 15 × 15 | 19 × 19 | 23 × 23 | 27 × 27 | 31 × 31 | 35 × 35 |
|  | $\sigma$^2)^ | 2.8 | 4.5 | 6.3 | 8.2 | 10.2 | 12.3 | 14.6 | 17.0 |
|  | $\lambda$^3)^ | 3.5 | 5.6 | 7.9 | 10.3 | 12.7 | 15.4 | 18.2 | 21.2 |
| Local max | Window size | 8 × 8 | 10 × 10 | 12 × 12 | 14 × 14 | 16 × 16 | 18 × 18 | 20 × 20 | 22 × 22 |
|  | Strides^4)^ | 4 | 5 | 6 | 7 | 8 | 9 | 10 | 11 |
| Area encoded by a single component | Size in pixel | 14 × 14 | 20 × 20 | 26 × 26 | 32 × 32 | 38 × 38 | 44 × 44 | 50 × 50 | 56 × 56 |
|  | Size in visual angle (°) | 1.4 × 1.4 | 2.0 × 2.0 | 2.6 × 2.6 | 3.2 × 3.2 | 3.8 × 3.8 | 4.4 × 4.4 | 5.0 × 5.0 | 5.6 × 5.6 |
